# Therapeutic effects of orexin-A in sepsis-associated encephalopathy in mice

**DOI:** 10.1186/s12974-024-03111-w

**Published:** 2024-05-17

**Authors:** Jing Guo, Zhuo Kong, Sha Yang, Jingjing Da, Liangzhao Chu, Guoqiang Han, Jian Liu, Ying Tan, Jiqin Zhang

**Affiliations:** 1 GuiZhou University Medical College, Guiyang, 550025 Guizhou Province China; 2 Department of Anesthesiology, Guizhou Provincial People’s Hospital, Guiyang, China; 3 Department of Neurosurgery, Guizhou Provincial People’s Hospital, Guiyang, China; 4 Department of Nephrology, Guizhou Provincial People’s Hospital, Guiyang, China; 5 Department of Neurosurgery, the Affiliated Hospital of Guizhou Medical University, Guiyang, China

**Keywords:** Orexin-A, Sepsis-associated encephalopathy, Inflammation, Cognitive dysfunction, Orexin receptors

## Abstract

**Background:**

Sepsis-associated encephalopathy (SAE) causes acute and long-term cognitive deficits. However, information on the prevention and treatment of cognitive dysfunction after sepsis is limited. The neuropeptide orexin-A (OXA) has been shown to play a protective role against neurological diseases by modulating the inflammatory response through the activation of OXR1 and OXR2 receptors. However, the role of OXA in mediating the neuroprotective effects of SAE has not yet been reported.

**Methods:**

A mouse model of SAE was induced using cecal ligation perforation (CLP) and treated via intranasal administration of exogenous OXA after surgery. Mouse survival, in addition to cognitive and anxiety behaviors, were assessed. Changes in neurons, cerebral edema, blood-brain barrier (BBB) permeability, and brain ultrastructure were monitored. Levels of pro-inflammatory factors (IL-1β, TNF-α) and microglial activation were also measured. The underlying molecular mechanisms were investigated by proteomics analysis and western blotting.

**Results:**

Intranasal OXA treatment reduced mortality, ameliorated cognitive and emotional deficits, and attenuated cerebral edema, BBB disruption, and ultrastructural brain damage in mice. In addition, OXA significantly reduced the expression of the pro-inflammatory factors IL-1β and TNF-α, and inhibited microglial activation. In addition, OXA downregulated the expression of the Rras and RAS proteins, and reduced the phosphorylation of P-38 and JNK, thus inhibiting activation of the MAPK pathway. JNJ-10,397,049 (an OXR2 blocker) reversed the effect of OXA, whereas SB-334,867 (an OXR1 blocker) did not.

**Conclusion:**

This study demonstrated that the intranasal administration of moderate amounts of OXA protects the BBB and inhibits the activation of the OXR2/RAS/MAPK pathway to attenuate the outcome of SAE, suggesting that OXA may be a promising therapeutic approach for the management of SAE.

**Supplementary Information:**

The online version contains supplementary material available at 10.1186/s12974-024-03111-w.

## Introduction

Sepsis is a systemic inflammatory response caused by infection or suspected infection, and is the leading cause of morbidity and mortality in intensive care units. Sepsis-associated encephalopathy (SAE) is defined as widespread brain dysfunction resulting from a systemic inflammatory response, and is characterized by disruption of the sleep-wake cycle, confusion, cognitive dysfunction, and coma [1]. SAE significantly impacts patient prognosis [2, 3], and studies have shown that sepsis survivors remain at risk of long-term sequelae, such as cognitive impairment and depression [4]. Clinical management primarily focuses on treating symptoms, and rapid fluid resuscitation is a key therapeutic strategy. However, there is a significant risk of complications including metabolic acidosis and hyperkalemia [5]. In recent years, several neuroprotective agents have been reported for SAE, including ginsenosides [6] and glucocorticoids [7]. However, information regarding the treatment of cognitive impairment in sepsis is sparse [8]. Therefore, developing effective treatment strategies for this condition is important.

The blood-brain barrier (BBB) comprises endothelial cells of the cerebral capillaries, pericytes, astrocytes, and tight junctions within the basement membrane [9], and serves as the brain’s defense mechanism against foreign pathogens and toxins in the blood [10]. In sepsis, the barrier function of the BBB is reduced or absent, allowing a large number of inflammatory factors and cells from the peripheral blood enter the brain tissue through the damaged or collapsed BBB, leading to brain damage [11]. As such, mitigation of BBB damage plays an important role in improving the prognosis of SAE.

The primary mechanism underlying sepsis is an uncontrolled inflammatory response. As the body’s inflammatory cascade response develops, a significant number of inflammatory cells accumulate at the site of injury, causing functional damage to various organs, including the brain tissue [4]. Blocking microglial activation and reducing the production of toxic factors after activation may facilitate SAE treatment [12, 13]. As such, the rational modulation of inflammation is critical for optimizing the outcomes of SAE [14–16], while effective drugs to reduce the incidence and severity of infectious brain injury are necessary.

Recent studies have shown that the nervous system directly influences the course of SAE via the production of neuropeptides [17]. Orexin is an excitatory neuropeptide synthesized and secreted by the hypothalamus. Orexin is classified into two types: orexin A (OXA) and orexin B (OXB). OXA and OXB bind to the G protein-coupled receptors orexin-1 receptor (OXR1) and orexin-2 receptor (OXR2), respectively, to regulate several important physiological activities in organisms [18]. OXA has the same affinity for OXR1 and OXR2, whereas OXB preferentially binds to OXR2 [19]. Because OXB is rapidly degraded in the circulation following peripheral administration, the intact form of OXB cannot be detected in the brain, while OXA can cross the BBB mainly by simple diffusion [20]. Intranasal administration has been validated as a delivery strategy to allow rapidly delivery of OXA to the CNS with minimal systemic exposure [21]. Given this information, in the present study, we focused on OXA administered intranasally. A large number of previous studies have shown that an imbalance in OXA is associated with Parkinson’s disease [22], Alzheimer’s disease [23], and other neurological disorders. Animal studies have further shown that OXA reduces neuroinflammation [24, 25], inhibits the endothelial cell inflammation induced by oxidized low-density lipoproteins in endothelial cells [26], and inhibits neuronal apoptosis [27]. Thus, OXA may be a potential therapeutic drug for central nervous system cognitive disorders. However, no studies have yet confirmed the therapeutic effects and exact mechanism of action of OXA on SAE.

In the present study, we found that OXA had a therapeutic effect on cecal ligation perforation (CLP)-induced pathological injury and cognitive dysfunction in SAE, and elucidated that the mechanism may be related to BBB protection and the inhibition of OXR2/RAS/MAPK pathway activation. Our findings not only provide neuropeptide candidates for the treatment of SAE, but also provide important insights into the pathological mechanisms and potential drug targets.

## Materials and methods

### Animals

Male C57BL/6J mice (6–8 weeks old, 20–25 g) were purchased from Tengxin Biotech (Chongqing, China). All mice were maintained at 22 ± 2 °C under a 12-h light/dark cycle with water and food ad libitum. This animal study was approved by the Guizhou Provincial Institutional Animal Care and Use Committee of the Provincial People’s Hospital (Guizhou, China).

### Preparation of CLP-induced septic mouse model

The modelling method was based on previous protocols [28, 29]. In brief, mice were anesthetized by sevoflurane inhalation. After depilation, the abdomen was sterilized and a longitudinal incision of approximately 1.0 cm was made along the midline. The cecum was carefully removed with sterile forceps, ligated with sterile 5 − 0 silk, and perforated at the blind end with a 22G injection needle to avoid damage to the mesentery and cecal vessels. After a small amount of fecal matter was expelled by gentle squeezing of the cecum, it was returned to the abdominal cavity. The Sham group underwent the same procedure but without ligation and perforation. Finally, the abdominal incision was closed in layers, and all mice were injected intraperitoneally with saline (5 ml/100 g) to replenish the body fluids lost during surgery.

### Administration

OXA (HY-106,224, MedChemExpress) was dissolved in saline and the OXA drug solution was administered intranasally. OXA was administered at three dose levels of 50 µg/kg (low dose), 250 µg/kg (medium dose), and 500 µg/kg (high dose) [30], 30 min after CLP induction [31], in a volume of 2.5 µl per nostril (5.0 µl per mouse). In the OXA optimal dose experiment, mice were intranasally administered OXA 250 µg/kg once daily for 7 days.

SB-334,867 (HY-10,895, MedChemExpress) [32, 33] and JNJ-10,397,049 (HY-108,906, MedChemExpress) [34], which are selective antagonists for OXR1 and OXR2, respectively, were dissolved in 5% DMSO to form a drug solution (10 mg/ml). Thirty minutes before CLP induction, 100 µL of the solution was injected intraperitoneally (i.p.), while the control group received the same volume (100 µl) of 5% DMSO.

#### Open field test (OFT)

The OFT is a classic behavioral test used to assess spontaneous activity, anxiety, and exploratory behavior in animals. This test was performed 1 and 7 days after surgery. The experimental apparatus comprised an opaque white cubic box measuring 50 × 50 × 40 cm. Each mouse was placed in a box with its back on one side of the box wall, and allowed to explore freely for 5 min. The total distance travelled and time spent were recorded using an automatic video tracking system (RWD Life Science Co. Ltd., China). The apparatus was wiped with 75% alcohol to remove odors.

In the OFT, the total distance travelled and the time spent in the central area were assessed.

### Y-maze test (Y-maze)

The Y-maze test is primarily used to test discriminative learning and working memory in animals. It consists of three white opaque arms, each measuring 30 × 5 × 20 cm (RWD Life Science Co., Ltd., China). In our experiments, one arm was chosen as the “start arm,” another as the “novel arm” and the third as the “other arm.” The animal was first placed in the “start arm” with its back to the center, after which the “novel arm” was closed. The animals were allowed to explore freely between the arms for 15 min during the training session. One hour later, the animal was placed in the “start arm” and then returned to the center. The “novel arm” was activated and the animals were allowed five minutes to explore freely. The time spent by the mice on each arm and the distance travelled by the animals on each arm were recorded. These measurements were used as indices to assess spatial recognition and memory. The maze was cleaned with 75% ethanol solution before each test.

In the Y-maze test, exploration and memory skills were quantified as the percentage of time spent exploring the new arm: %time = (zone C activity time/test time) × 100. We further assessed the percentage of distance travelled within the new arm of the activity as follows: %travel distance = (zone C distance/total distance) × 100.

### Tail suspension test (TST)

The TST was used to evaluate depression-related behavior. This test was carried out one and seven days after surgery, according to a previously established method [35]. In a dark and quiet room, the mice were taped approximately 1 cm from the tip of their tails and suspended upside down with their heads approximately 30 cm above the top of the table. The resting time of each mouse was recorded for 5 min.

In the TST, anxiety-like behavior was quantified as the total time the mice remained stationary for 5 min.

#### Determination of brain water content

The mice were euthanized after surgery. The brain was immediately excised and the wet weight was determined It was then dried at 100 °C for 24 h and weighed again to determine the dry weight. The water content of brain tissue was then calculated. The formula for calculating the percentage water content of brain tissue is: (wet weight of brain tissue - dry weight of brain tissue) / wet weight × 100%.

### BBB permeability

#### Assessment of Evans blue dye extravasation

A 2% Evans blue (EB) solution (Sigma-Aldrich) was dissolved in saline and injected into the caudal vein at a dose of 4 ml/kg. After 1 h, all the animals were euthanized and transcardially perfused with 0.9% saline for 20 min to remove the intravascular dye. The hippocampus was subsequently isolated from the brain, homogenized with 0.5 ml of 60% trichloroacetic acid and incubated at 4 °C for 30 min. After centrifugation (12,000 rpm for 15 min), absorbance was measured at 620 nm using an enzyme marker (BioTek, USA).

### Endogenous immunohistological detection of IgG extravasation

Mice were anesthetized, cardiac perfusion was performed, and the brains were removed and fixed in 4% paraformaldehyde (PFA) at 4 °C for over 24 h. Sections of 20 μm thickness were then taken using a frozen section machine (Leica, Japan). The cells were then treated with Alexa Fluor 488-conjugated goat anti-mouse IgG (A-32,723, Invitrogen) for 1 h. The fluorescence intensity of IgG was determined using a research slide scanner (Zeiss, LSM 980).

### Immunofluorescence (IF) staining

Mouse brain tissue was cryosectioned and incubated with 10% goat serum (G1208, Servicebio). Slices were then incubated with rabbit anti-Iba-1 (EPR16588, Abcam) overnight at 4 °C, and the samples were subsequently treated with an Alexa Fluor Plus 594-conjugated goat anti-rabbit fluorescent secondary antibody (A-11,062, Invitrogen) for 1 h. Finally, fluorescence images were captured using a laser scanning confocal microscope (Zeiss, LSM 980) and analyzed using ImageJ software.

### Western blotting

Hippocampal tissue was homogenized and centrifuged, and the supernatant was extracted. Proteins were extracted according to the manufacturer’s instructions and quantified using a BCA protein assay kit (Well-Bio, China). Equal amounts of protein (30 µg) were separated on 10% acrylamide gels, and transferred to 0.22 μm PVDF membranes. After blocking, primary antibodies diluted in the antibody diluent were added for incubation at 4 °C overnight and then washed. The following primary antibodies were used: rabbit anti-Rras (ER60170, HuaBio), rabbit anti-Orexin-A (BS15009R, Bioss), rabbit anti-Orexin receptor 2 (Ab183072, Abcam), rabbit anti-Orexin receptor 1 (#59,242), rabbit anti-Ras (#3965), rabbit anti-IL-1β (#31,202), rabbit anti-phospho-MAPK family (#9926) and MAPK family (#9926) (Cell Signaling Technology), rabbit anti-TNF-α (A11534, Abclonal). Horseradish peroxidase (HRP)-conjugated secondary antibody (GB23303, Servicebio) was added, and the mixture was washed three times at room temperature. The results were analyzed using the ImageJ software.

### Observation of ultrastructural changes by transmission electron microscopy

Seven days after model establishment, mice were anesthetized and perfused with glutaraldehyde fixative, and the hippocampus was rapidly removed and immersed in a 2% glutaraldehyde solution for more than 24 h. They were then fixed in 4% glutaraldehyde for 2 h, rinsed thrice with PBS, and fixed in 1% osmium for 2 h. Tissues were dehydrated using an acetone gradient, polymerized at 37 °C and 60 °C in a thermostat, and sectioned at a thickness of approximately 50 nm using an ultrathin sectioning machine. Finally, the cell ultrastructure was observed by transmission electron microscopy after ultrathin double staining with uranyl acetate and lead acid.

### Nissl staining

Mouse brains were collected 1 day after surgery to assess hippocampal damage. The hearts were perfused with 4% paraformaldehyde-PBS solution and fixed in 4% paraformaldehyde solution for 24 h, then paraffin-embedded before taking 5 μm thick coronal sections. The sections were placed in Nissl staining solution (G1036, Servicebio) and neurons in the CA1 area of the hippocampus were observed under a light microscope (Olympus, Japan).

### Quantitative proteomic analysis

Hippocampal tissues from the brains of mice in the sham, CLP, and OXA optimal dose (250 µg/kg) groups were rapidly harvested 7 days after surgery and stored in liquid nitrogen. Proteomic identification was performed by Hangzhou Jingjie Biotechnology Co. on nine mice (three mice per group). Samples were collected for protein concentration using a BCA kit. Samples were then desalted on a Strata X C18 solid-phase extraction column (Phenomenex) and dried under vacuum. Separation was performed using high-pH reversed-phase high-performance liquid chromatography. Tryptophan peptides were treated and applied to an NSI source before being subjected to tandem mass spectrometry (MS/MS) using a Q Exactive™Plus (Thermo Scientific, Waltham).

### Enzyme-linked immunosorbent assay

Commercial ELISA kits (mouse TNF-α, EK0527, Boster and mouse IL-1β, EK0394, Boster) were used. After diluting the standard, samples were added vertically to each well without touching the wall of the well. Then, 50 µl of enzyme reagent was added, and the plate was sealed with a membrane for incubation at 37 ºC for 30 min. Subsequently, the development solution was added to develop color, and the reaction was finally terminated. The optical density at 450 nm was analyzed using a spectrophotometer, and the protein concentration was quantified by comparing the OD of the target proteins with a standard curve.

### Statistical analysis

Data were processed and analyzed using GraphPad Prism software (version 9.0). All measurements were expressed as the mean ± standard deviation (X ± SEM). Statistical significance was calculated for univariate comparisons between three or more groups using one-way ANOVA followed by Tukey’s post-hoc test. *P <* 0.05 were considered statistically significant.

## Results

### OXA reduces neurocognitive/mood deficits and hippocampal neuronal apoptosis 1 day after sepsis in a dose-dependent manner

**Fig. 1:**
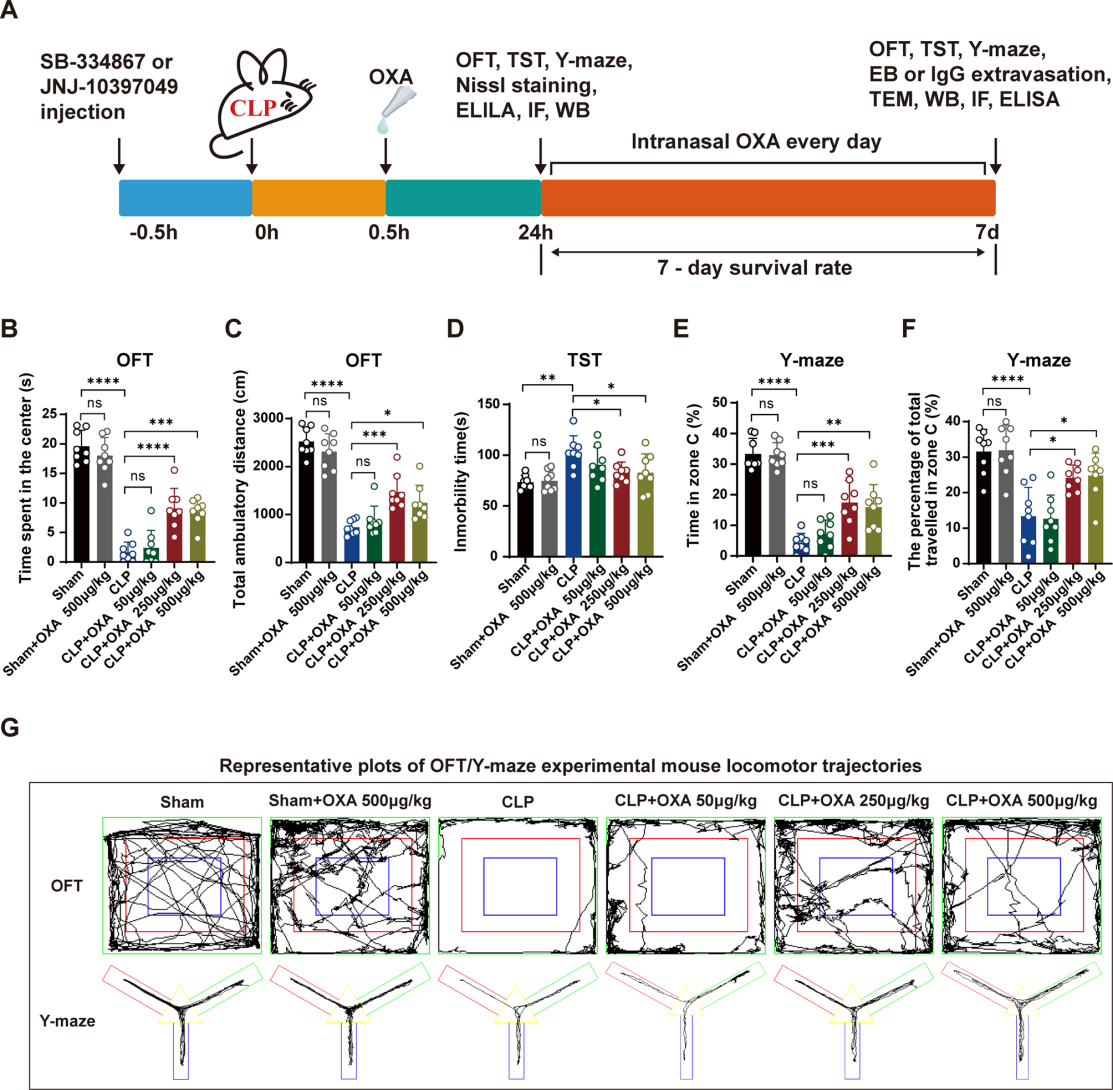
OXA ameliorates mood and cognitive deficits and hippocampal neuronal damage 1 day after sepsis in a dose-dependent manner. **A** Schematic timeline of the experimental procedures. **B** Time in the central zone and **C** distance of activity in the open field. **D** Resting time within 5 min in the tail suspension test. **E** Percentage of time spent exploring and **F** percentage of distance activity in the C-arm in the Y-maze. **G** Open field test and Y-maze movement trajectory maps. The above behavioral tests were performed 1 day after surgery. *n* = 8 per group. Data are presented as mean ± SEM. *p < 0.05, **p < 0.01, ***p < 0.001, ****P < 0.0001

To assess whether the CLP-induced sepsis mouse model exhibited cognitive dysfunction, we performed behavioral tests and evaluated the effects of OXA on any observed deficiencies. Mice were assessed for cognitive, motor, and emotional deficits using the OFT. As shown in Fig. 1B, C, there were no significant differences in the proportion of distance travelled or time spent in the central area between the sham and sham + OXA 500 µg/kg groups. CLP mice showed a significant decrease in central area dwell time and distance travelled (****p < 0.0001, **** *p* < 0.0001 vs. sham group), while OXA-treated mice in the CLP + OXA 250 µg/kg and CLP + OXA 500 µg/kg groups had significantly increased central area dwell time and distance travelled (****p < 0.0001, ***p < 0.001, ***p < 0.001, *p < 0.05 vs. CLP group). However, compared with the CLP group, the difference was not statistically significant in the CLP + OXA 50 µg/kg group.

Mood disturbance was assessed using the TST. As shown in Fig. 1D, there were no significant differences in the proportion of resting time after tail suspension within 5 min between the sham and sham + OXA 500 µg/kg groups. CLP mice showed a significant increase in resting time within 5 min (**p < 0.01 vs. sham group), OXA-treated mice in the CLP + OXA 250 µg/kg and CLP + OXA 500 µg/kg groups showed a decreased resting time (*p < 0.05, *p < 0.05 vs. CLP). However, compared with the CLP group, the difference was not statistically significant in the CLP + OXA 50 µg/kg group.

The exploratory and memory abilities of mice were assessed using the Y-maze test. As shown in Fig. 1E, F, there were no significant differences in the percentage of exploration time and activity distance within the new arm (C-arm) of the mice between the sham and sham + OXA 500 µg/kg groups. The percentage of exploration time and activity distance was significantly decreased in CLP mice (****p < 0.0001, ****p < 0.0001 vs. sham group), while OXA-treated mice in the CLP + OXA 250 µg/kg and CLP + OXA 500 µg/kg groups had increased percentage of exploration time and percentage of distance (***p < 0.001, *p < 0.05, **p < 0.01, *p < 0.05 vs. CLP group). However, compared with the CLP group, the difference was not statistically significant in the CLP + OXA (50 µg/kg) group.

Pathological changes of neurons in the CA1 area of the hippocampus were observed by Nissle staining. Overall, neurons in the sham and sham + 500 µg/kg OXA groups were neatly and compactly arranged, with rounded cells and obvious rounded nuclei in multilayered formations. Conversely, neurons in the CLP and low dose OXA (50 µg/kg) groups were vacuolated, disorganized and sparsely arranged with irregular morphology. Neuronal damage was ameliorated in the OXA medium (250 µg/kg) and high dose (500 µg/kg) groups (Figure S1).

The above results showed that one day after CLP, mice showed reduced basal motor activity, lack of exploration, increased anxiety-like behavior, and damage to hippocampal neurons. However, intranasal OXA showed protective effects and the most significant benefit was observed at a dose of 250 µg/kg. Therefore, future studies used 250 µg/kg OXA.

### Continuous administration of OXA for 7 days was effective at reducing mortality and improving cognitive and affective impairment after sepsis

**Fig. 2:**
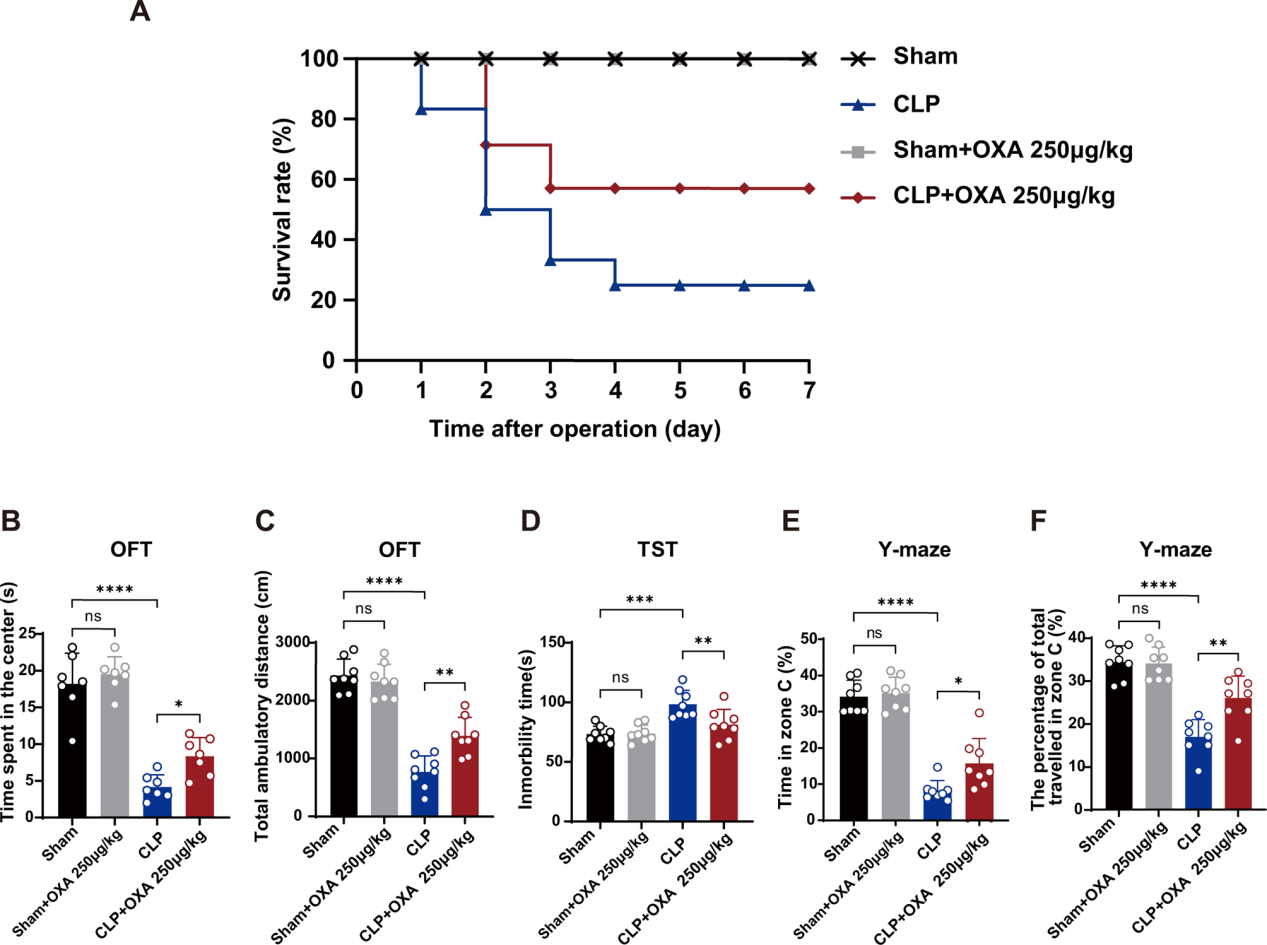
Treatment with 250 µg/kg OXA increases 7-day survival and ameliorates cognitive and affective deficits in CLP-induced sepsis. **A** Survival curves. Values are expressed as the survival rate. Overall, 8 out of 28 mice in the CLP group and 13 out of 23 mice in the OXA group survived to day 7. Surviving mice were used for subsequent experiments. **B** Time in the central zone and **C** distance of activity in the open field. **D** Resting time within 5 min in the tail suspension test. **E** Percentage of time exploration and **F** percentage of distance activity in the C-arm in the Y-maze. The above behavioral tests were performed 7 days after surgery. *n* = 8 per group. Data are presented as mean ± SEM. *p < 0.05, **p < 0.01, ***p < 0.001, ****p < 0.0001

Based on the results of the post-operative test, the dose of 250 µg/kg was used as the optimal dose for the subsequent test. To understand the effect of 250 µg/kg OXA on survival within 7 days after sepsis-induced brain injury, we assessed the mortality rate following daily intranasal administration OXA 250 µg/kg after CLP. All mice in the Sham group and the Sham + 250 µg/kg OXA group survived over 7 days. However, the 7-day survival rate of mice in the CLP group was low, with only 28% (8/28 mice) surviving; after treatment with OXA, the survival rate increased to 56% (13/23 mice). We found that 250 µg/kg OXA significantly increased the survival rate of mice with sepsis (Fig. 2A).

To further determine the improvement of 250 µg/kg OXA on cognitive and emotional deficits in mice after septic encephalopathy, we performed behavioral tests on mice 7 days after surgery. In the OFT, as shown in Fig. 2B, C, there were no significant differences in the proportion of distance travelled or time spent in the central region between the Sham and Sham + OXA 250 µg/kg groups. Conversely, CLP mice showed a significant decrease in the time spent in the central region and the distance travelled (****p < 0.0001, ****p < 0.0001 vs. sham group). The OXA group showed an increase in the distance travelled and time spent in the central region (*p < 0.05, **p < 0.01, vs. CLP group).

In the TST, as shown in Fig. 2D, there was no significant difference in resting time after tail suspension within 5 min between mice in the sham and sham + OXA 250 µg/kg groups. CLP mice showed a significant increase in resting time within 5 min (***p < 0.001 vs. sham group), and resting time was decreased in the OXA group (**p < 0.01 vs. CLP group).

In the Y-maze, as shown in Fig. 2E, F, there were no significant differences in the percentage of exploration time and activity distance within the new arm (C-arm) of the mice between the sham and sham + OXA 250 µg/kg groups. The percentage of exploration time and activity distance were significantly reduced in CLP mice (****p < 0.0001, ****p < 0.0001 vs. sham group). The percentage of exploration time within the new arm and the percentage of active distance increased in the OXA group (*p < 0.05, **p < 0.01, vs. CLP group).

These results suggested that OXA is effective at reducing mortality and ameliorating cognitive and affective deficits in postseptic mice, leading us to investigate the underlying protective mechanisms.

### OXA ameliorates BBB disruption in SAE mice

**Fig. 3:**
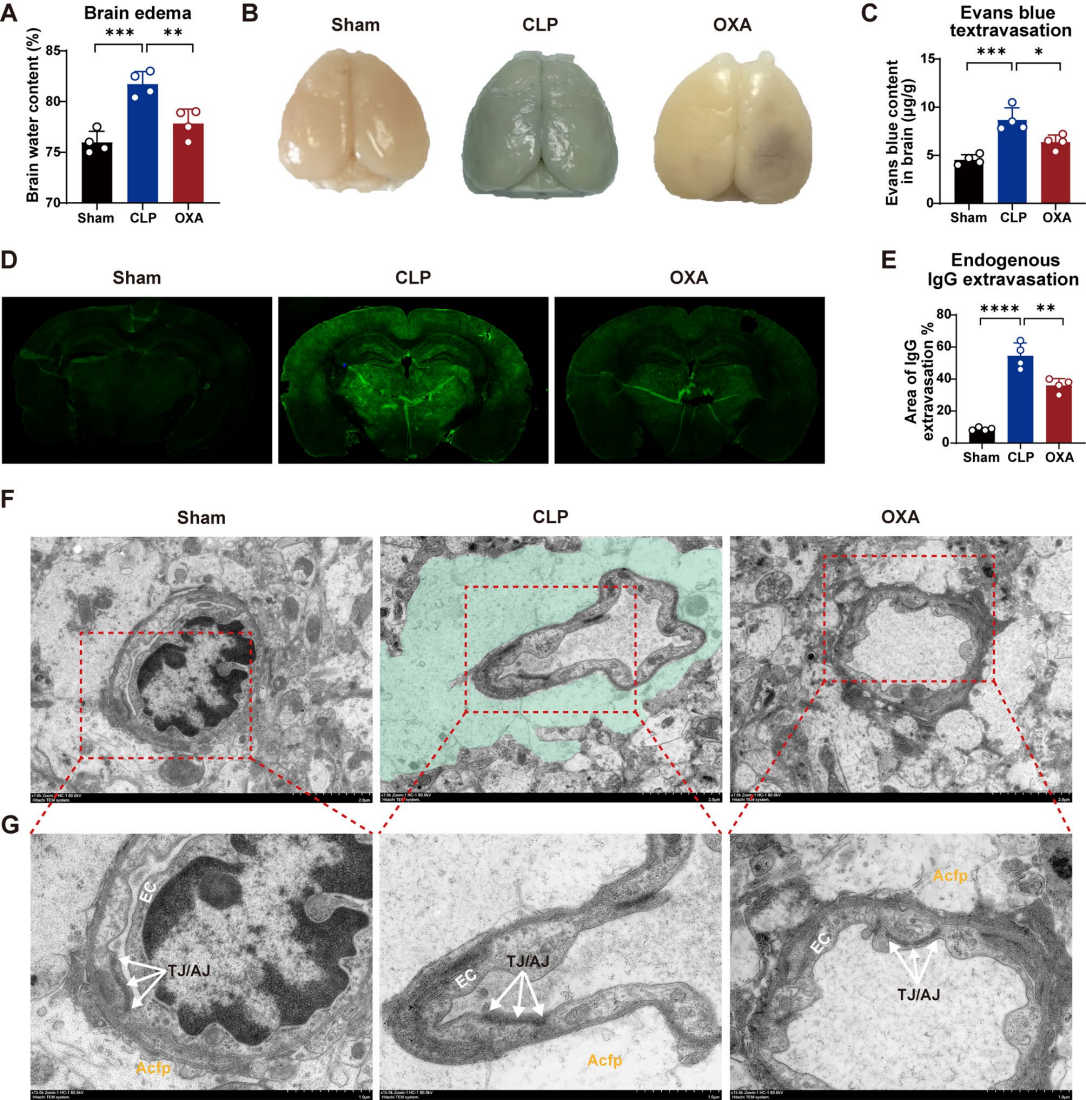
OXA attenuates cerebral edema, protects BBB integrity, and ameliorates ultrastructural damage 7 days after CLP. **A** Content of cerebral edema. **B**, **C** Extravasation of EB. **D**, **E** Extravasation of IgG. OXA treatment reduced extravasation of endogenous IgG into brain tissue. *n* = 4 per group. **F** Representative transmission electron microscopy images showing the BBB ultrastructure. In the CLP group, the edematous astrocyte endfeet (teal) enwrap the vessel. Scale bar = 2 μm. *n* = 4 per group. **G** G is a magnified view of F. Tight junctions of the BBB are highlighted at high magnification. TJ/AJ = tight and adherens junctions; EC = brain endothelial cells; Acfp = astrocyte foot processes. Scale bar = 1 μm

The BBB ensures normal brain function by maintaining the stability of the brain microenvironment [36]. To investigate whether OXA had a protective effect on the BBB, we first examined the degree of cerebral edema. As shown in Fig. 3A, the brain water content increased in the CLP group (***p < 0.001 vs. sham group) and decreased in the OXA group (**p < 0.01 vs. CLP group). Subsequently, we evaluated the permeability of the BBB. As shown in Fig. 3B and C, in contrast to the sham group, the EB content in the brain tissue significantly increased in the CLP group (***p < 0.001 vs. sham group), and OXA significantly decreased the EB content (*p < 0.05, vs. CLP group). IgG in the brain is predominantly secreted by immune cells that infiltrate the brain; therefore, we examined the level of IgG in the brain. The data in Fig. 3D and E show that OXA administration after CLP significantly decreased the area of IgG penetration in the brain (**p < 0.01 vs. CLP group). These data indicated that OXA improved BBB integrity after CLP.

Next, we examined the BBB ultrastructure. Electron microscopy of brain microvessels in the hippocampus of the mouse brain 7 days after surgery revealed that the inner layer of the vessel wall in the sham group consisted of endothelial cells, the outer layer consisted of pericytes, and the outermost layer appeared as a footplate for processes derived from astrocytes surrounding the periphery (Fig. 3F). In the CLP group, the blood vessels were significantly constricted and the vessel lumen collapsed. The endfeet of astrocytes attached to the blood vessels were also significantly swollen. The swollen areas (teals) were bright and hyaline. However, in the OXA-treated group, endothelial cells and basement membrane structure remained intact, indicating an improvement in structural damage. We found no obvious differences in the length or intensity of tight and adherens junctions (TJ/AJ) among the three groups (Fig. 3G). Furthermore, electron microscopy of astrocytes revealed significant cellular edema after CLP. Astrocytes with medium-sized nuclei and homogeneous cytoplasmic structures were observed in both the Sham and OXA-treated groups. However, the cytoplasm of the model group was slightly stained and appeared hyaline, whereas fragments of different sizes and large and small vesicles due to cellular edema were visible. Significant damage to the organelles were observed (Figure S2). Overall, these results suggest that the BBB is disrupted after CLP, but that OXA treatment ameliorates BBB damage.

### OXA improves ultrastructural damage in septic mice

**Fig. 4:**
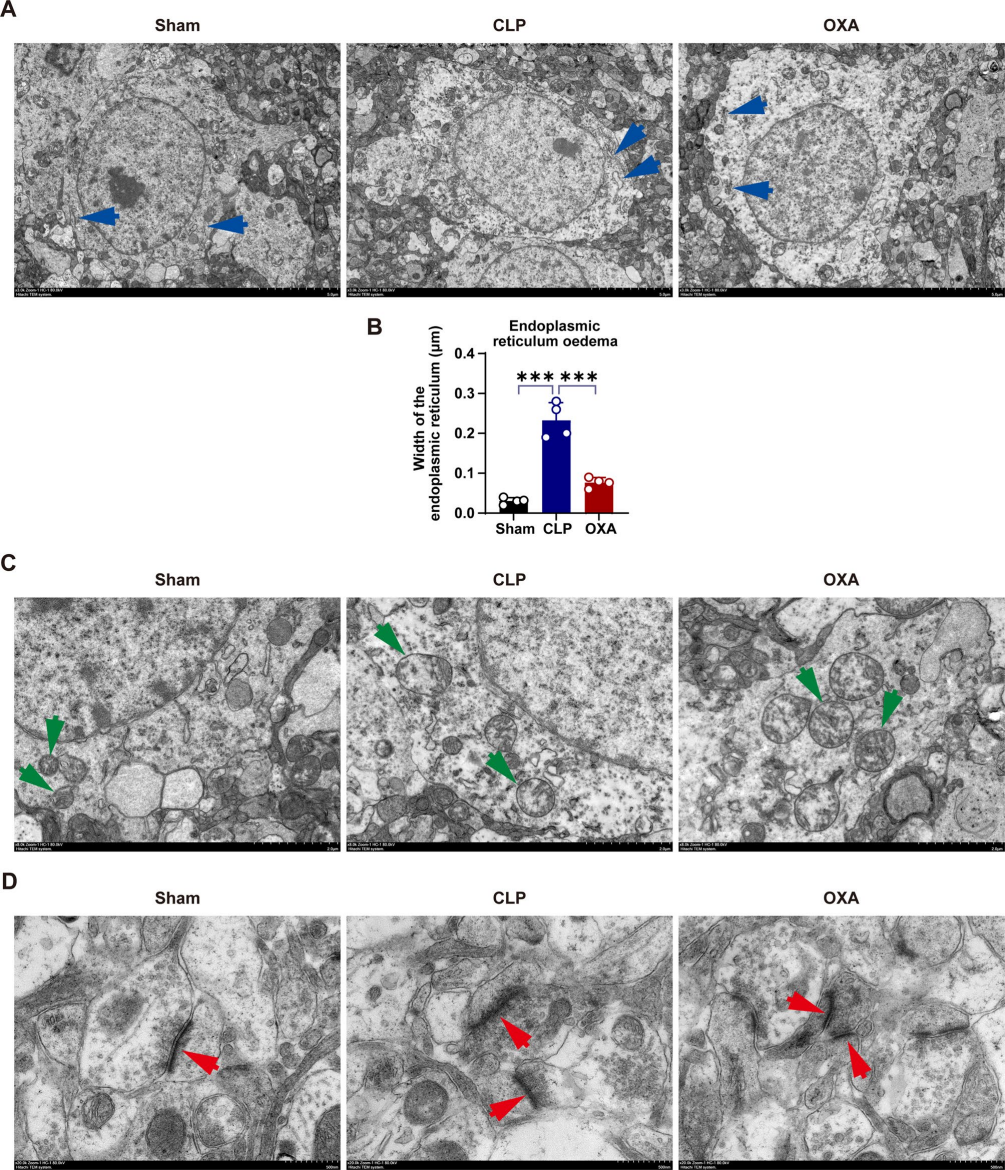
OXA treatment for 7 days attenuates ultrastructural damage in septic mice. **A** Representative transmission electron micrograph of three groups of hippocampal neurons, blue arrow: endoplasmic reticulum. Scale bar = 5 μm. B Quantification of endoplasmic reticulum width in neurons. Values represent the mean of each group. *n* = 4 per group. **C** C is a magnified view of A. High magnification of neurons showing details of mitochondria, green arrows: mitochondria. Scale bar = 2 μm. **D** Representative electron micrographs of three groups of hippocampal synapses, red arrows: synaptic structures. *n* = 4 per group. Scale bar = 500 nm. Data are presented as mean ± SEM. *p < 0.05, **p < 0.01, ***p < 0.001, ****P < 0.0001

We observed the ultrastructural characteristics of the brains in mice with sepsis to assess the brain protective effects of OXA. The results showed that the endoplasmic reticulum is significantly altered. As shown in Fig. 4A and B, the hippocampal endoplasmic reticulum of mice in the CLP group showed significant edema and dilation (***p < 0.001 vs. sham group), which improved in the OXA-treated group (***p < 0.001 vs. CLP group). The mitochondria in the CLP group were swollen and the cristae were weakened or even disappeared, whereas the mitochondrial structure was improved in the OXA group (Fig. 4C). The synaptic structure observed using electron microscopy showed clear gaps between the presynaptic and postsynaptic membranes in the sham group. However, the CLP group showed significant fusion between the presynaptic and postsynaptic membranes, as well as damage and an indistinct diffuse gap in the postsynaptic membrane. In the OXA-treated group, the presynaptic membrane was partially fused with the postsynaptic membrane, resulting in less damage (Fig. 4D). These results suggested that treatment with OXA ameliorated the ultrastructural damage induced by CLP in the brains of mice with sepsis.

### OXA reduces septic encephalopathy-induced inflammation and inhibits microglial activation in the cortex and hippocampus of mice

**Fig. 5:**
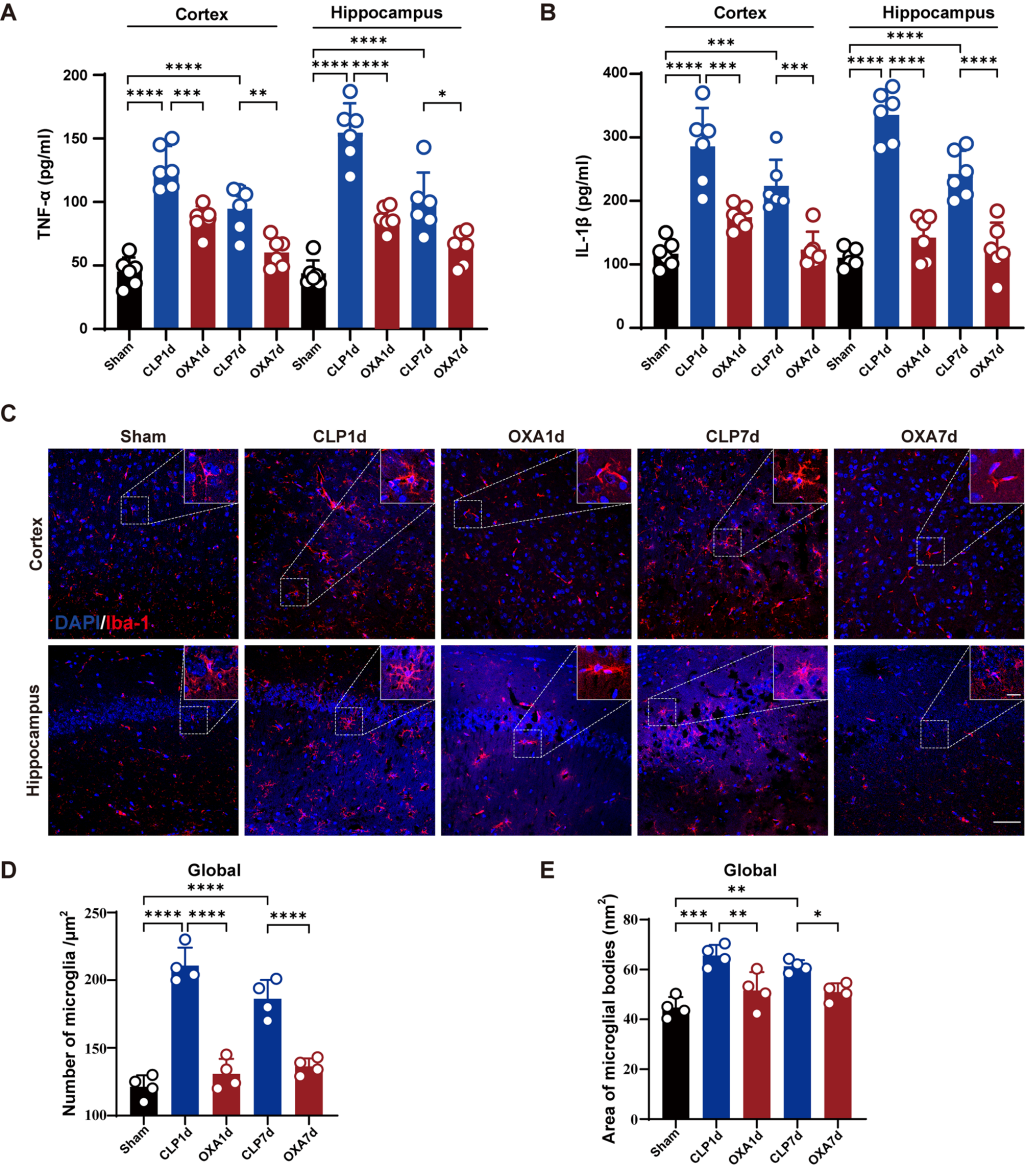
OXA reduces inflammatory cytokine levels and microglial activation in CLP-induced SAE. **A**, **B** Expression levels of TNF-α and IL-1β in cortical and hippocampal tissue. *n* = 6 per group. **C** Cortex and hippocampus with representative Iba-1 and DAPI immunofluorescence staining. Scale bar = 100 μm. The upper right corner shows representative magnified Iba-1-positive cells. Scale bar = 20 μm. **D**, **E** Whole brain Iba-1 positive cell counts and statistical analysis of the cytosolic area of positive cells were performed. *n* = 4 per group, with 4 sections per animal. Data are presented as mean ± SEM. *p < 0.05, **p < 0.01, ***p < 0.001, ****P < 0.0001

Sepsis is an inflammatory disease that affects the immune system. The release of inflammatory factors exacerbates inflammatory response and apoptosis, leading to brain tissue damage. As shown in Fig. 5A, B, ELISA revealed a significant increase in TNF-α and IL-1β levels in cortical and hippocampal tissues on both the first and seventh day after sepsis induction (****p < 0.0001, ****p < 0.0001, ****p < 0.0001, ****p < 0.0001 vs. sham group), OXA treatment significantly reduced the expression levels of TNF-α and IL-1β (***p < 0.001, **p < 0.01, ****p < 0.0001, *p < 0.05, ***p < 0.001, ****p < 0.0001 vs. CLP group).

Microglial activation mediates neuroinflammation in the brain. Microglial immunoreactivity in the hippocampus and cortex was assessed using Iba-1 staining. As shown in Fig. 5C and D, immunofluorescence images of the hippocampus and cortex were taken on the first and seventh days after sepsis, showing a significant increase in the number of Iba-1-positive cells (****p < 0.0001, ****p < 0.0001 vs. sham group), while the number of activated microglia in the cortex and hippocampus of the OXA-treated group was significantly reduced (****p < 0.0001, ****p < 0.0001 vs. CLP group). Similarly, magnified images of Iba1-positive cells in septic mice showed an enlarged cell body and thick processes, consistent with activated microglia (***p < 0.001, **p < 0.01 vs. sham group). In contrast, microglial cells in the OXA group had thin cell bodies and elongated processes, consistent with the morphology of resting microglial cells (**p < 0.01, *p < 0.05 vs. CLP group).

### Proteomic sequencing analysis

**Fig. 6:**
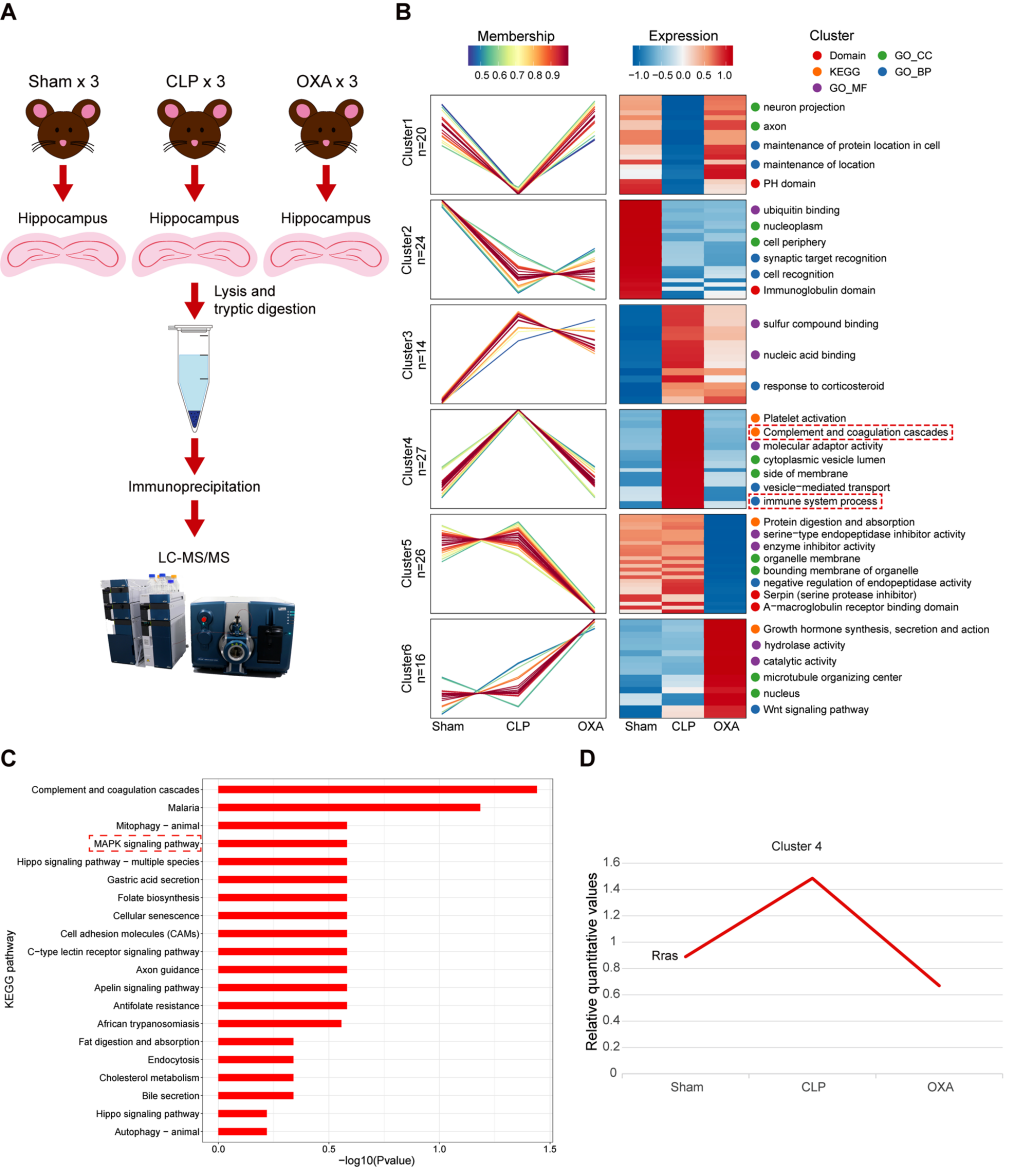
Proteomic clustering analysis of hippocampal tissue in the mouse brain. **A** Pattern diagram of the proteomic analysis process. **B** Clustered protein expression patterns in the Sham, CLP, and OXA groups. Six clusters were identified by co-expression. Each fold line represents one protein. Left panel: Co-expression patterns of proteins in the six clusters. Right panel: The top two most significantly enriched entries in each cluster resulting from the enrichment analysis. **C** KEGG enriched pathway of proteins in cluster 4. **D** Quantitative expression of Rras proteins in the MAPK pathway in cluster 4. *n* = 3 per group

To investigate the specific mechanism by which OXA exerts anti-inflammatory effects in septic brain injury, we collected hippocampal tissue from the sham, CLP, and OXA (250 µg/kg) groups on the 7th day after surgery. Samples were subjected to enzymatic digestion and tandem LC-MS/MS analysis. Bioinformatic tools were used to perform quantitative proteomic analysis (Fig. 6A), identifying a total of 5680 proteins. To identify proteins that underwent significant dynamic changes after OXA treatment, we performed expression pattern clustering analysis. Relative protein expression was first transformed using a Log2 logarithmic transformation. Proteins with an SD > 0.5 were then screened. After screening, the remaining 127 proteins were used for cluster analysis based on their expression patterns. The number of clusters, k, was set to six, and the degree of cluster fuzziness, m, was set to two. As shown in Fig. 6B, the expression pattern classes (clusters) corresponding to the labelled proteins, with proteins in the same cluster showing similar trends in expression transformation, were divided into six groups. Statistical analysis revealed significant upregulation of protein expression levels in the fourth cluster of proteins after CLP. Furthermore, it was observed that the protein expression levels were restored after treatment with OXA. This finding suggests that the fourth cluster of proteins might play a critical role in the rescue or regulatory effects of OXA treatment. Therefore, we focused on investigating specific proteins within this cluster and their relationship with the therapeutic effects of the drug. GO and KEGG analyses of cluster 4 proteins indicated that their molecular functions were primarily associated with complement and coagulation cascades, as well as immune response processes. This further suggests that the inhibition of the inflammatory response may be an important mechanism underlying the treatment of CLP-induced SAE.

Specific KEGG pathways enriched in cluster 4 proteins included complement and coagulation cascades, malaria, and MAPK (Fig. 6C). The MAPK pathway is critical for cell proliferation, stress responses, and inflammation. It activates microglia to release inflammatory factors, leading to inflammation and apoptosis in the central nervous system [37, 38]. Therefore, we focused on the MAPK pathway, and the expression of Rras proteins in cluster 4 of the MAPK pathway was quantitatively measured to create a line graph (Fig. 6D). Rras is a member of the small G protein superfamily known as RAS. RAS is a key protein involved in the MAPK signaling pathway which acts as a molecular switch interacting with downstream factors to transduce extracellular signals into the intracellular environment [39]. We examined the expression of the Rras and the RAS/MAPK axis in the sham, CLP, and OXA groups.

### OXA reduces the expression of the Rras protein and inhibits the activation of the RAS/MAPK pathway to reduce the expression of inflammatory factors

**Fig. 7:**
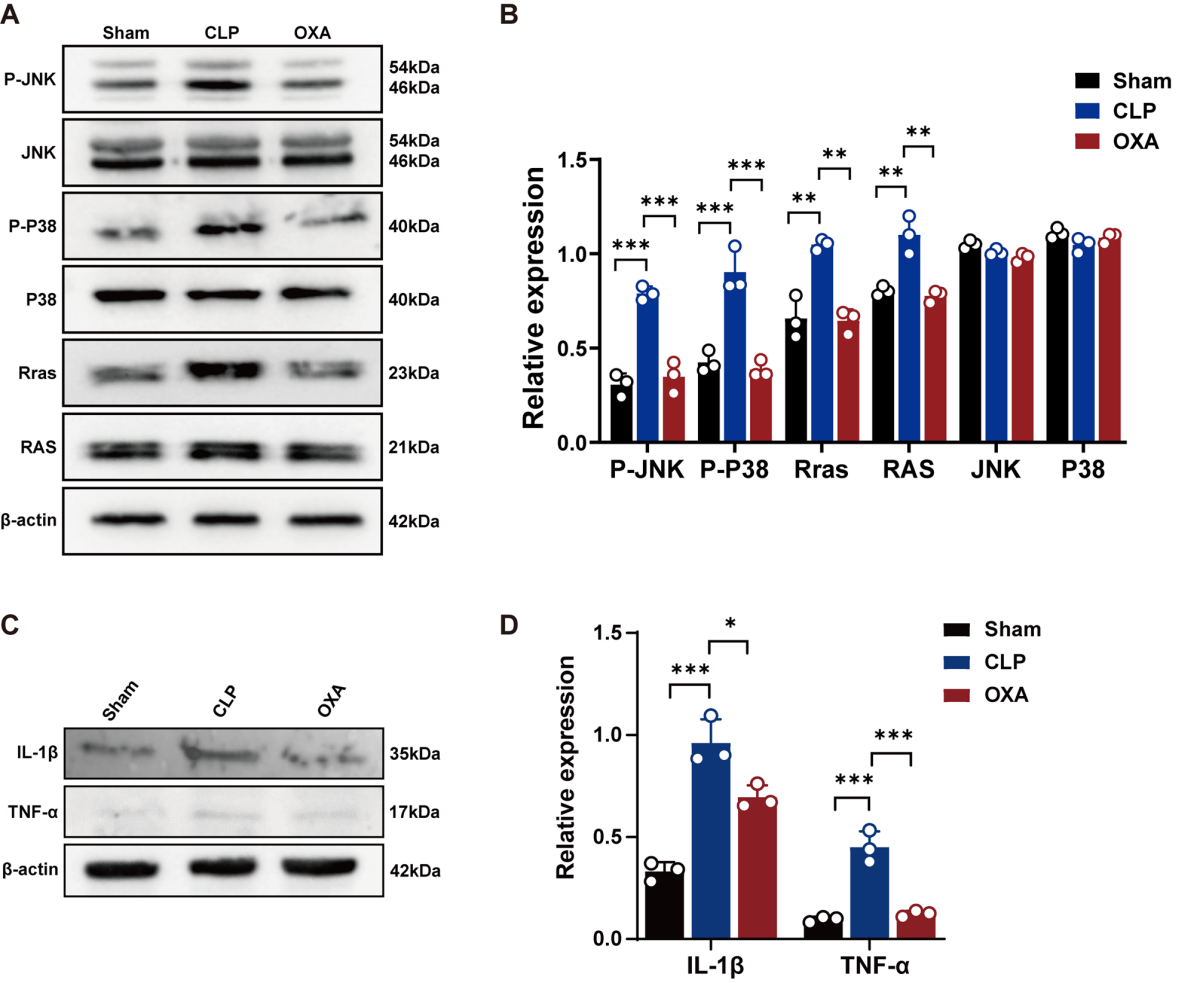
OXA reduces inflammation by decreasing Rras expression and impeding activation of the RAS/MAPK pathway. **A**, **B** Representative protein bands and quantitative analysis of Rras, RAS, P-JNK/JNK, P-P38/P38 and **C**, **D** IL-1β, TNF-α in hippocampal tissue 7 days after modelling. *n* = 3 per group. Data are presented as mean ± SEM. *p < 0.05, **p < 0.01, ***p < 0.001, ****p < 0.0001

To validate the proteomic results, we examined the protein expression levels of the Rras and the RAS/MAPK axis in the hippocampal tissues of mice in the sham, CLP, and OXA groups. Western blot analysis was performed for verification. As shown in Fig. 7A and B, the results showed that the expression of Rras, RAS, P-JNK and P-P38 significantly increased after CLP (**p < 0.01, **p < 0.01, ***p < 0.001, ***p < 0.001 vs. sham group), and the phosphorylation levels of Rras, RAS, P-JNK and P-P38 significantly decreased after OXA administration (**p < 0.01, **p < 0.01, ***p < 0.001, ***p < 0.001 vs. CLP group). As shown in Fig. 7C, D, the expression of downstream inflammatory factors IL-1β and TNF-α was also increased in the model group (***p < 0.001, ***p < 0.001 vs. sham group), while OXA treatment led to a decrease in the expression of inflammatory factors (*p < 0.05, ***p < 0.001 vs. CLP group). Based on the above studies, we hypothesized that the neuroprotective mechanism of OXA after CLP involves a reduction in inflammation by decreasing Rras protein expression and inhibiting the activation of the RAS/MAPK pathway.

### Differential effects of OXR1 and OXR2 receptors in the regulation of inflammatory expression through the MAPK pathway

**Fig. 8:**
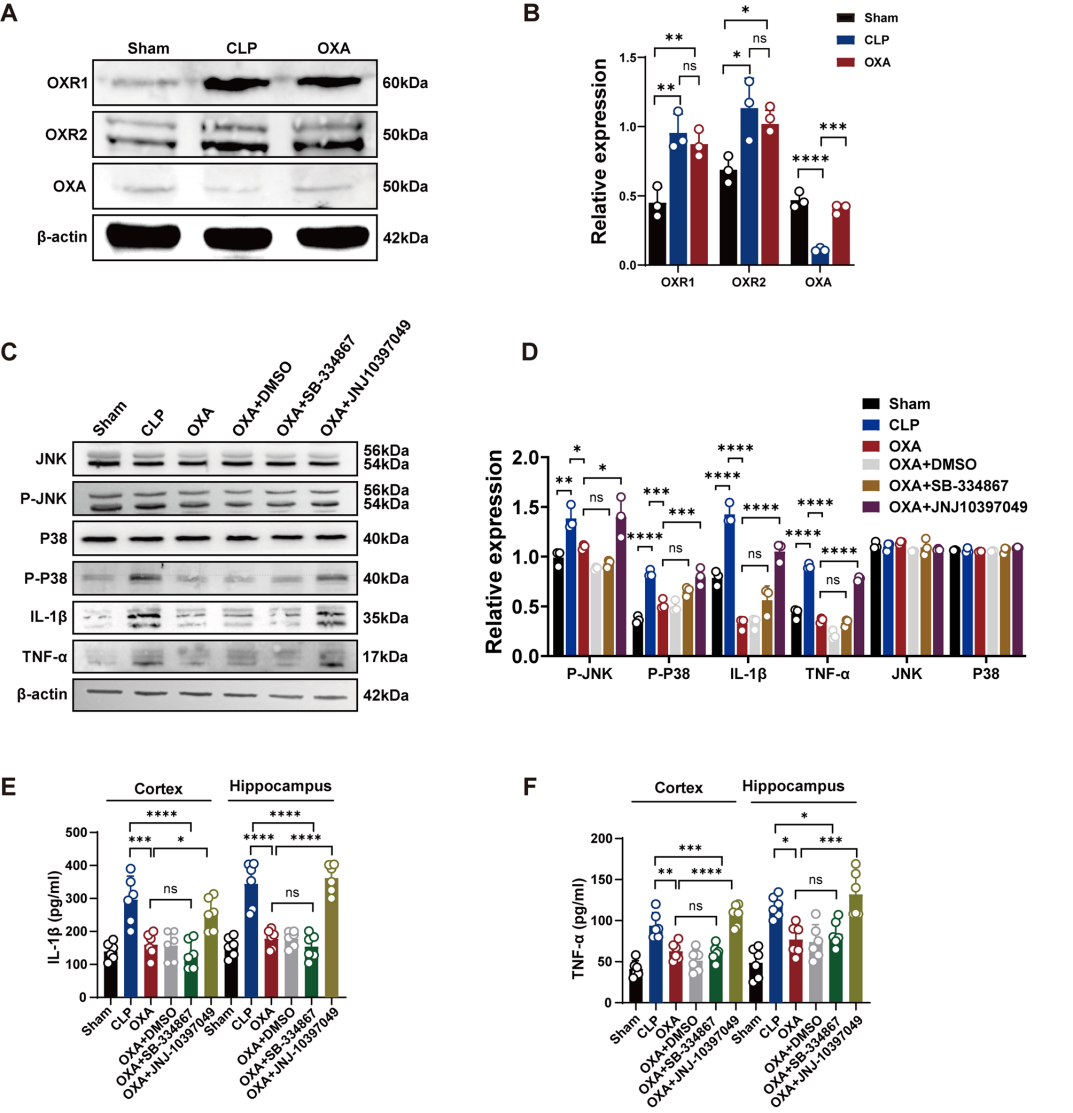
Differences in the roles of OXR1 and OXR2. **A**, **B** Representative protein bands and quantitative analysis for OXR1, OXR2, and OXA in hippocampal tissue 24 h after CLP. **C**, **D** Representative protein bands and quantitative analysis of P-JNK, P-P38, IL-1β, and TNF-α after administration of SB-334,867 (OXR1 blocker) or JNJ10397049 (OXR2 blocker) 24 h after CLP. *n* = 3 per group. **E**, **F** Levels of IL-1β and TNF-α in brain tissue 24 h after CLP. *n* = 6 per group. Data are presented as mean ± SEM. *p < 0.05, **p < 0.01, ***p < 0.001, ****P < 0.0001

Exogenous OXA expression in the brain was detected by western blotting (Fig. 8A). Protein blot analysis showed that OXA expression was reduced in the hippocampal tissue of the mouse brain after CLP (****p < 0.0001 vs. sham group). However, OXA protein levels significantly increased after OXA administration (***p < 0.001 vs. CLP group), and as shown in Fig. 8A, B, the expression of OXR1 and OXR2 expression was not affected by OXA treatment.

As shown in Fig. 8C, D, consistent with the findings of previous studies, OXA treatment significantly decreased the expression of P-JNK, P-P38 and the downstream inflammatory factors IL-1β and TNF-α (**p < 0.01, ****p < 0.0001, ****p < 0.0001, ****p < 0.0001 vs. CLP group). These effects were not observed when a selective OXR1 inhibitor (SB-334,867) was administered. However, administration of the selective OXR2 inhibitor (JNJ-10,397,049) significantly increased the expression of P-JNK, P-P38 and the downstream inflammatory factors IL-1β and TNF-α in mouse hippocampal tissue (*p < 0.05, ***p < 0.001, ****p < 0.0001, ****p < 0.0001 vs. CLP + OXA group).

**Fig. 9:**
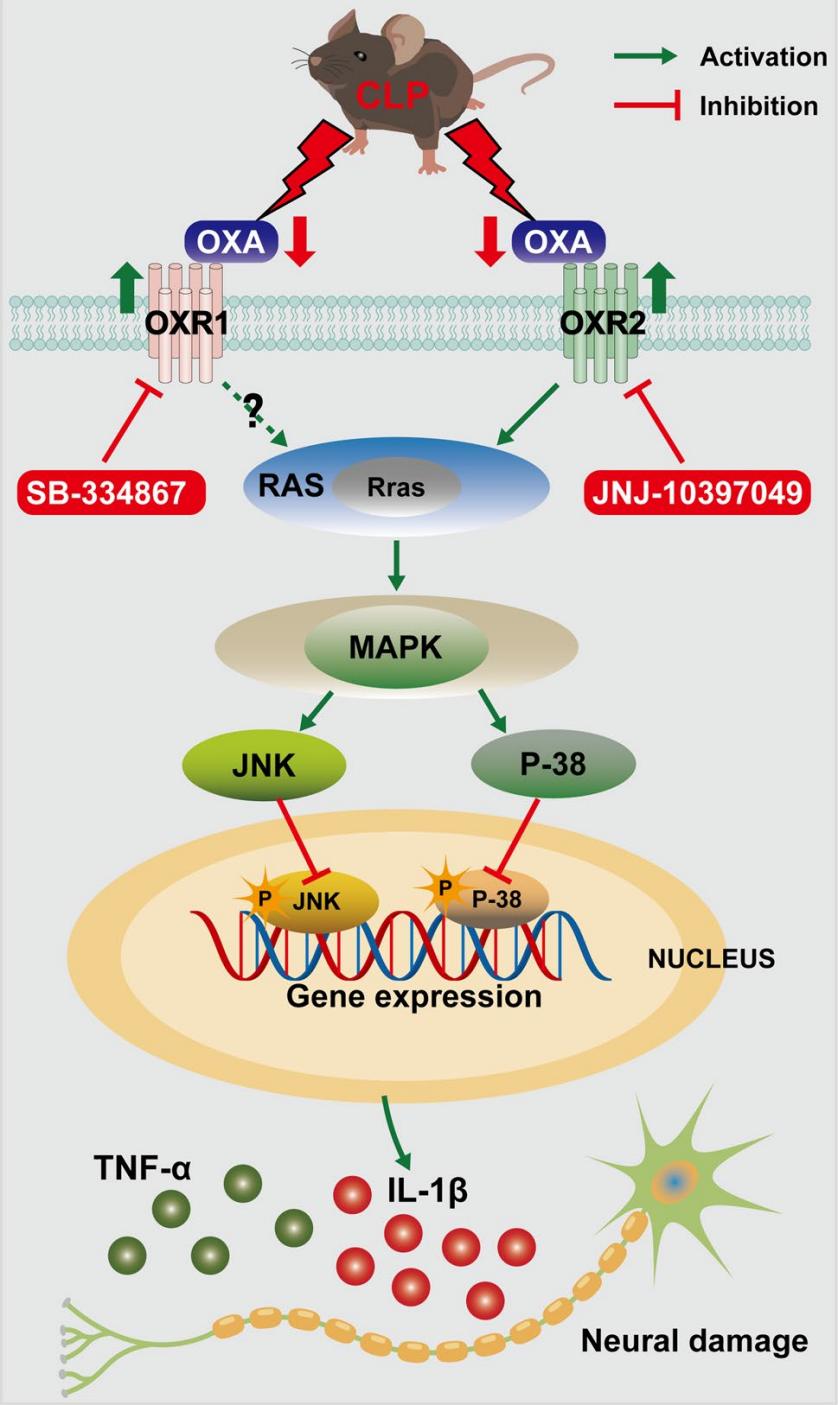
Anti-inflammatory signaling pathway mediated by OXA and OXR2 in SAE

Furthermore, the anti-inflammatory mechanisms of OXA and its receptors were evaluated by ELISA. As shown in Fig. 8E, F, the expression levels of IL-1β, TNF-α were significantly downregulated in the cortical and hippocampal tissues after OXA treatment (***p < 0.001, ****p < 0.0001, **p < 0.01, *p < 0.05 vs. CLP group). The effects of OXA were significantly reversed by an OXR2 inhibitor (JNJ10397049) (*p < 0.05, ****p < 0.0001, ****p < 0.0001, ***p < 0.001 vs. OXA group), but could not be reversed by an OXR1 inhibitor (SB-334,867). These results suggest that the anti-inflammatory effect of OXA on SAE is mediated mainly by OXR2 (Fig. 9).

## Discussion

The pathogenesis of clinical SAE is characterized by altered mental status, with a wide range of neurological pathologies, ranging from delirium to coma, impaired cognitive performance and consciousness, and episodes of inattention and depression [4, 40]. There is growing evidence to suggest that the high mortality rate of sepsis is closely related to acute or long-term cognitive impairment [41, 42]. The hippocampus plays a prominent role in the cognitive and emotional responses. The CLP model is one of the most prominent models for investigating sepsis and related complications, and its ability to mimic perforated appendicitis in clinical patients makes it the gold standard of animal models for studying sepsis [29]. Xu [43] and others [44] have both previously conducted behavioral studies in septic mice, stating that the mice exhibited cognitive dysfunction and anxiety after CLP. Consistent with previous studies, we observed that, on the day after the model was established, the mice showed obvious erect hair, shortness of breath, purulent secretions from the eyes, anorexia, and unresponsiveness. Meanwhile, on the seventh day after CLP, the mice showed obvious autonomous exploration, decreased spatial memory, and anxiety-like behavior. Expansion of the endoplasmic reticulum, swelling of mitochondria, marked disruption of cristae, as well as fusion of presynaptic and postsynaptic membranes, as well as other manifestations of acute cellular injury in neurons of septic mice were observed by electron microscopy, which may underlie the pathology of cognitive dysfunction, and evidence from the literature supports such a hypothesis [45–47].

LPS-induced SAE may indirectly inhibit orexigenic activity through an indirect mechanism [48–50]. Some investigators have found a 6-fold decrease in appetitive hormone activity after CLP in mice [51]. These studies further demonstrated the relevance and importance of appetitive hormone activity in the pathogenesis of sepsis. Intranasal (IN) drug delivery has been reported to provide a rapid and direct route to the central nervous system [52, 53], and allows safe and noninvasive targeting of peptide drugs to the brain [54, 55]. The precise route of drug delivery from the nose to the brain has been demonstrated, mainly involving the systemic, olfactory, and trigeminal pathways [56, 57], with delivery via the systemic route exhibiting characteristics similar to intravenous injection [58, 59]. Studies have reported that intranasal administration of OXA improves neurofunctional outcomes and reduces cerebral edema following cerebral hemorrhage [60]. Thus, IN-orexin may be a promising noninvasive treatment strategy for cognitive decline [61]. However, few studies have investigated the effects of orexin ingestion on cognitive function. In the present study, we investigated the optimal dosage for effective OXA treatment of CLP-induced SAE-like brain injury. We investigated the effects of three different doses of OXA (50 µg/kg, 250 µg/kg, and 500 µg/kg) on cognitive function, finding that OXA showed a dose-dependent improvement in protecting against cognitive impairment caused by SAE. In addition, we confirmed that treatment with 250 µg/kg OXA significantly reduced mortality and ameliorated cognitive impairment by postoperative day 7 in SAE mice. Overall, we showed that OXA can effectively treat sepsis, and provide an optimal dose of the drug to ameliorate behavioral deficits.

At the onset of SAE, endothelial cell dysfunction leads to the impairment of the blood-CSF barrier, resulting in increased capillary permeability and vasodilation. This consequently leads to cerebral tissue ischemia and hypoxia [62]. Past studies have shown that BBB permeability increases in the cerebral cortex, hippocampus, and thalamus as early as 24 h after intraperitoneal injection of LPS [63]. This increase in BBB permeability may lead to the activation of neuroglia and production of cytotoxic mediators that act on the BBB, exacerbating injury. CLP causes significant cerebral edema, extravasation of EBs, and elevated IgG levels. IgG in the brain is primarily produced by the secretion of immune cells that infiltrate the brain. In this case, the immune system was clearly activated in sepsis; therefore, the cause of increased IgG levels was more likely to occur secondary to immune cell infiltration. Inflammation-induced ultrastructural changes in BBB cells remain poorly investigated; however, one of the few other studies investigating ultrastructural changes in the BBB revealed marked edema around the capillaries following LPS-induced injury, but no significant changes in tight junctions [64]. In comparison with previous research, we did not observe any significant changes in the length and strength of tight junctions using electron microscopy, but did note significant capillary collapse in the model group. In addition, the swelling of astrocytes in the BBB structure showed extensive edema; to further confirm this result, we examined astrocytes specifically, finding that the astrocyte endfeet encircle the entire vascular tree within the central nervous system. These results support a morphological basis for BBB leakage. Many neurological disorders are characterized by the loss of polarization of specific endfoot proteins, vascular dysregulation, and BBB breakdown. The role of astrocyte endfeet has been demonstrated in many of these conditions [65]. Our study fills several knowledge gaps by demonstrating that OXA treatment reverses sepsis-induced BBB disruption, which may be related to SAE-induced cerebral vascular endothelial cell dysfunction and reduced vascular endothelial cell inflammation [26, 66]. Amelioration of barrier defects may protect against SAE-induced neuronal damage [67].

Neuroinflammation is the primary cause of apoptosis and the subsequent development of brain dysfunction. Peripheral administration of OXA has been shown to reduce microglial activation to protect against multiple sclerosis [68]. In the present study, we observed high expression of the inflammatory factors TNF-α and IL-1β, as well as significant activation of microglia in the cortex and hippocampus in brain tissue on days 1 and 7 after CLP. In addition, prior research has shown that activated microglial cells are capable of producing TNF-α, ultimately leading to neuronal damage [69]. To understand the inflammatory pathways activated by OXA inhibition, proteomic analysis was performed to identify the proteins and associated pathways that play critical roles in regulating the anti-inflammatory effects of OXA.

Rras is a member of the Ras superfamily of small G-proteins, and Ras superfamily proteins regulate cell proliferation, differentiation and apoptosis [70]. Rras shares approximately 55% amino acid sequence homology with RAS and there is significant overlap between Rras and RAS-mediated signaling pathways [28]. Previous studies have shown that mutations in components of the RAS/MAPK pathway cause neurocognitive deficits [71]. In addition, the RAS/MAPK pathway plays an important role in cancer progression [72, 73]. In the present study, we confirmed that the protein levels of Rras, RAS, P-JNK, and P-P38 were significantly increased in the hippocampal tissue after the induction of sepsis in a mouse model of CLP. This suggests that the P-JNK and P-P38 pathways are activated, confirming the involvement of the MAPK pathway in the development of CLP-induced SAE. However, treatment with OXA significantly reduced the protein expression of Rras and RAS, as well as the phosphorylation levels of JNK and P38, resulting in a decrease in the levels of the downstream inflammatory factors TNF-α and IL-1β. Therefore, it is reasonable to conclude that OXA reduces the release and production of pro-inflammatory factors by downregulating RAS expression and inhibiting the P-JNK and P-P38 signaling pathways.

OXA attenuates cerebral edema induced by cerebral hemorrhage and reduces neuroinflammation via the OXR2/AMPK axis [60]. In contrast, in studies of ischemic stroke, OXA significantly improved neurological deficit scores and reduced inflammatory mediators, primarily through inhibition of the OXR1/NF-κB pathway [66]. To gain more insights into the primary pharmacological targets of OXA in the RAS/MAPK pathway in CLP-induced septic encephalopathy, we performed further experiments using different OXR1 and OXR2 receptor blockers. Our results were validated by western blotting and ELISA. After testing relevant samples, the OXR1 inhibitor SB-334,867 showed results similar to those of OXA; however, the OXR2 inhibitor JNJ10397049 significantly reversed the effects of OXA. This suggests that OXA primarily binds to OXR2. OXR1 and OXR2 may exert different effects in different models of OXA binding to different receptors. We speculate that these differences in efficacy may be due to the different models and the activation of different signaling pathways. Many studies have shown that OXR1 and OXR2 bind to other subunits to form dimers that activate different downstream signaling molecules [74, 75]. This result confirms our hypothesis.

The orexin system is involved in a variety of physiological functions, including the central regulation of pancreatic fluid secretion and gastrointestinal motility. Oku [76] reported that orexin neuropeptides improve intestinal barrier function, and that the brain can control intestinal permeability. In the present study, we investigated the effect of OXA on the nervous system following peripheral administration of OXA; however, the effects of OXA on the intestinal tract and its mechanism were not investigated. This could be an area that could be explored in depth in subsequent studies.

## Conclusions

In summary, we observed that intranasal OXA administration increased the survival rate of septic mice after seven days, attenuated pathological brain damage and cognitive dysfunction, attenuated BBB damage, inhibited neuronal apoptosis, and reduced the levels of inflammatory factors. In addition, the proteomic results suggested that the protective mechanism of OXA may be related to an induced reduction of Rras and RAS proteins, as well as the inhibition of MAPK pathway activation. Furthermore, we identified the specific drug targets of OXA that exert anti-inflammatory effects via the MAPK pathway. Overall, these results enrich the understanding of the specific molecular mechanisms underlying the neuroprotective effects of OXA in SAE brain injury, which may provide promising drug strategies and targets for the treatment of septic encephalopathy.

### Electronic supplementary material

Below is the link to the electronic supplementary material.

Supplementary Material 1

Supplementary Material 2

## Data Availability

All relevant data are included in the manuscript and supplementary files. Any further inquiries can be directed to the corresponding author.
