# Supplementary material for: Therapeutic effects of orexin-A in sepsis-associated encephalopathy in mice
Source: J Neuroinflammation. 2024 May 17;21:131. doi: 10.1186/s12974-024-03111-w (PMC11102217; doi:10.1186/s12974-024-03111-w)
Supplement: Supplementary file 1 — Supplementary Material 1 [file 12974_2024_3111_MOESM1_ESM.pdf]

Figure. 7A

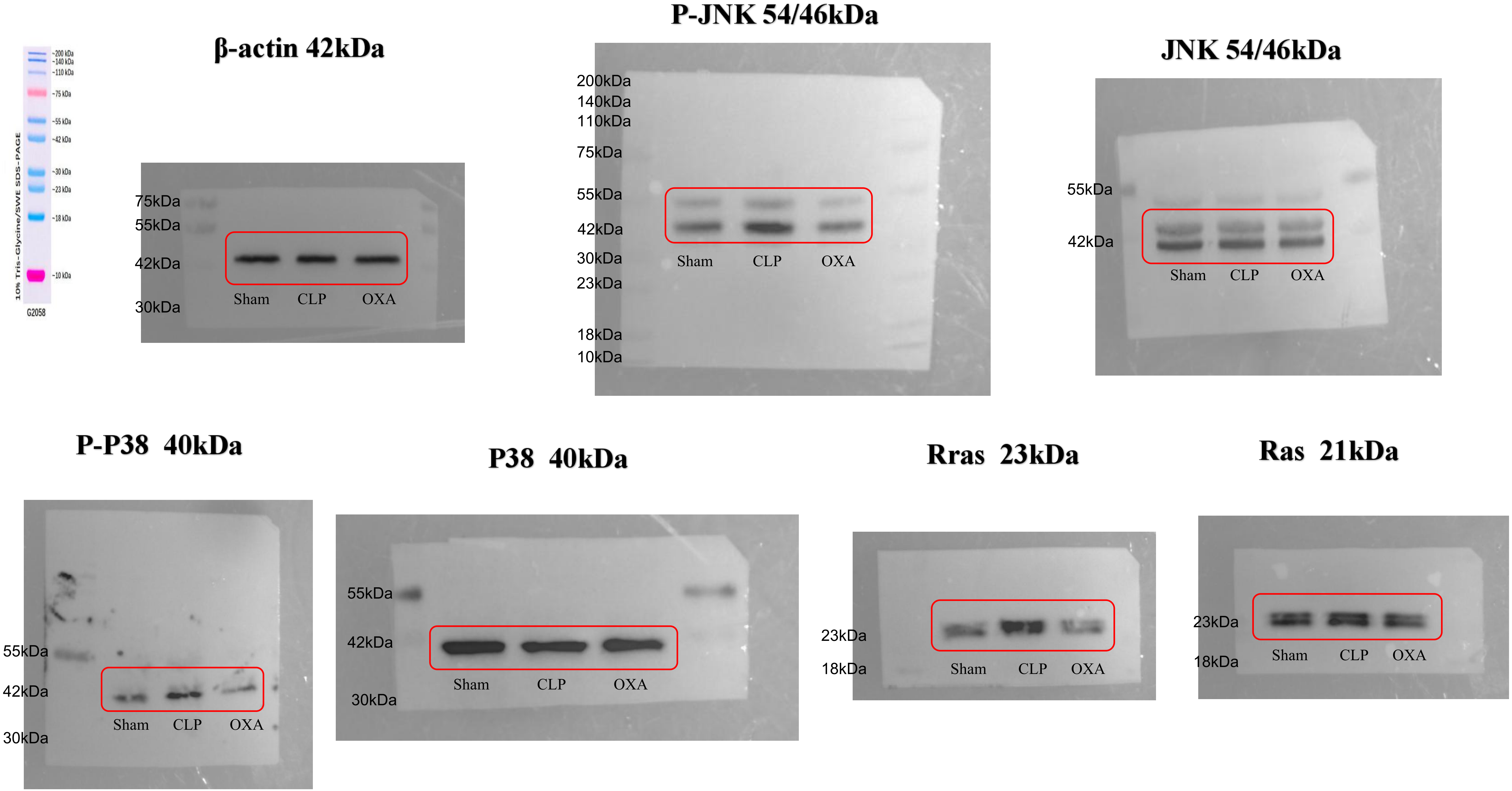
β-actin 42kDa
75kDa
55kDa
42kDa
30kDa
Sham CLP OXA
P-JNK 54/46kDa
200kDa
140kDa
110kDa
75kDa
55kDa
42kDa
30kDa
23kDa
18kDa
10kDa
Sham CLP OXA
JNK 54/46kDa
55kDa
42kDa
Sham CLP OXA
P-P38 40kDa
55kDa
42kDa
30kDa
Sham CLP OXA
P38 40kDa
55kDa
42kDa
30kDa
Sham CLP OXA
Rras 23kDa
23kDa
18kDa
Sham CLP OXA
Ras 21kDa
23kDa
18kDa
Sham CLP OXA

Figure. 7C

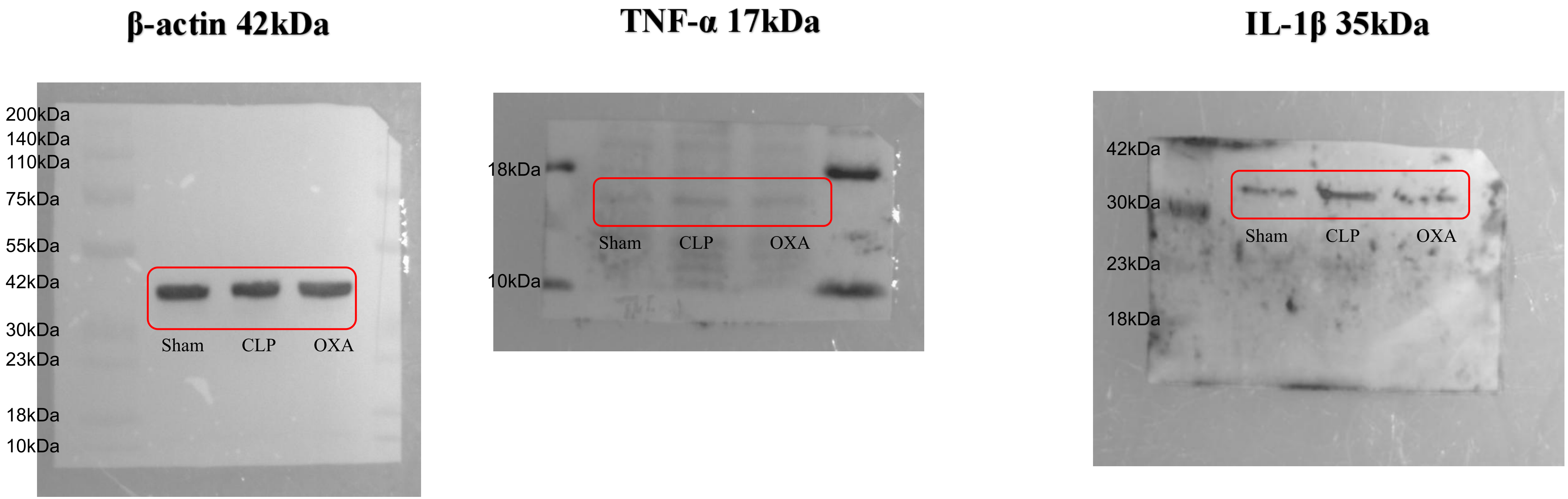
β-actin 42kDa
200kDa
140kDa
110kDa
75kDa
55kDa
42kDa
30kDa
23kDa
18kDa
10kDa
Sham
CLP
OXA
TNF-α 17kDa
18kDa
10kDa
Sham
CLP
OXA
IL-1β 35kDa
42kDa
30kDa
23kDa
18kDa
Sham
CLP
OXA

Figure. 8A

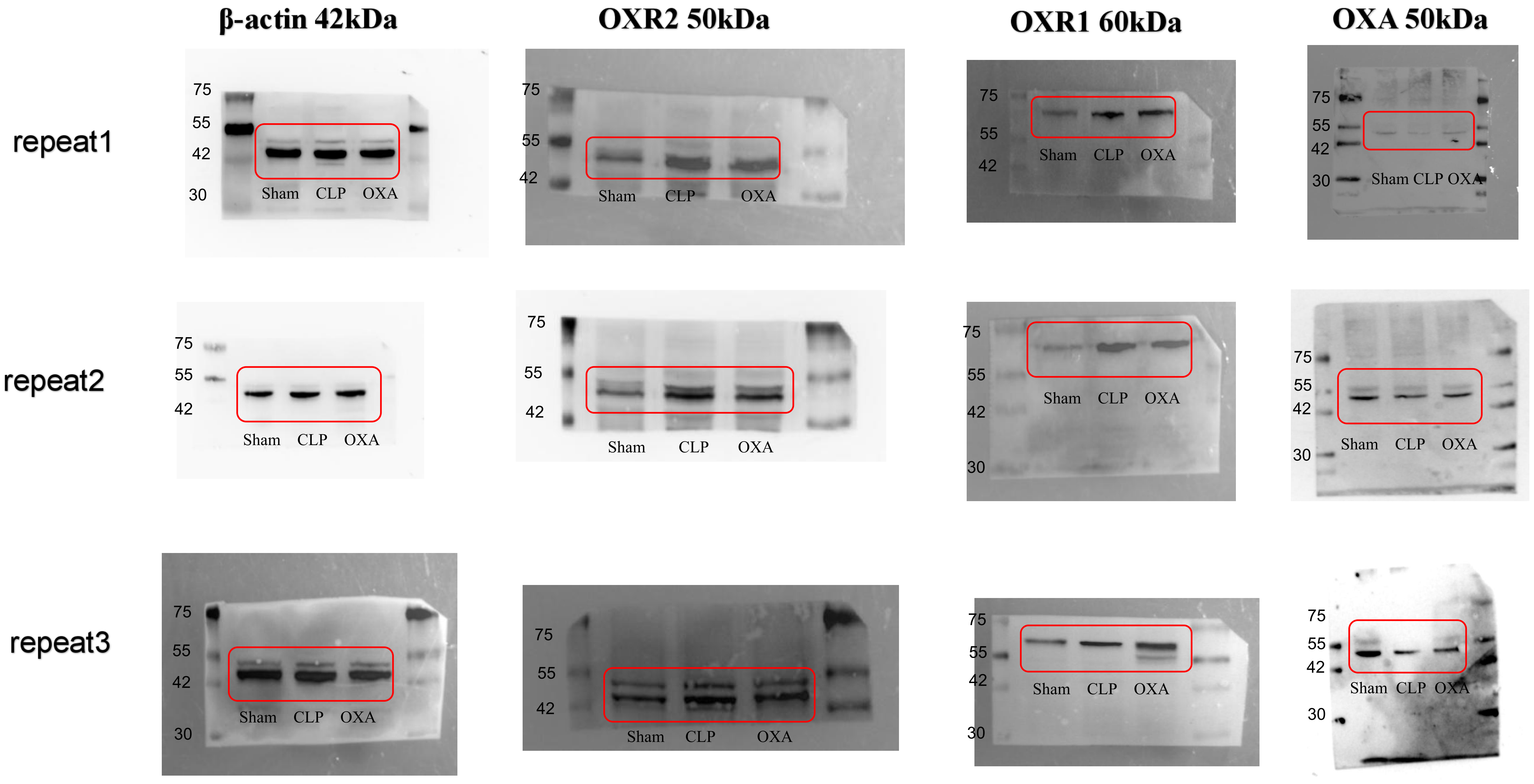
β-actin 42kDa
OXR2 50kDa
OXR1 60kDa
OXA 50kDa
repeat1
repeat2
repeat3
75
55
42
30
Sham
CLP
OXA

Figure. 8C

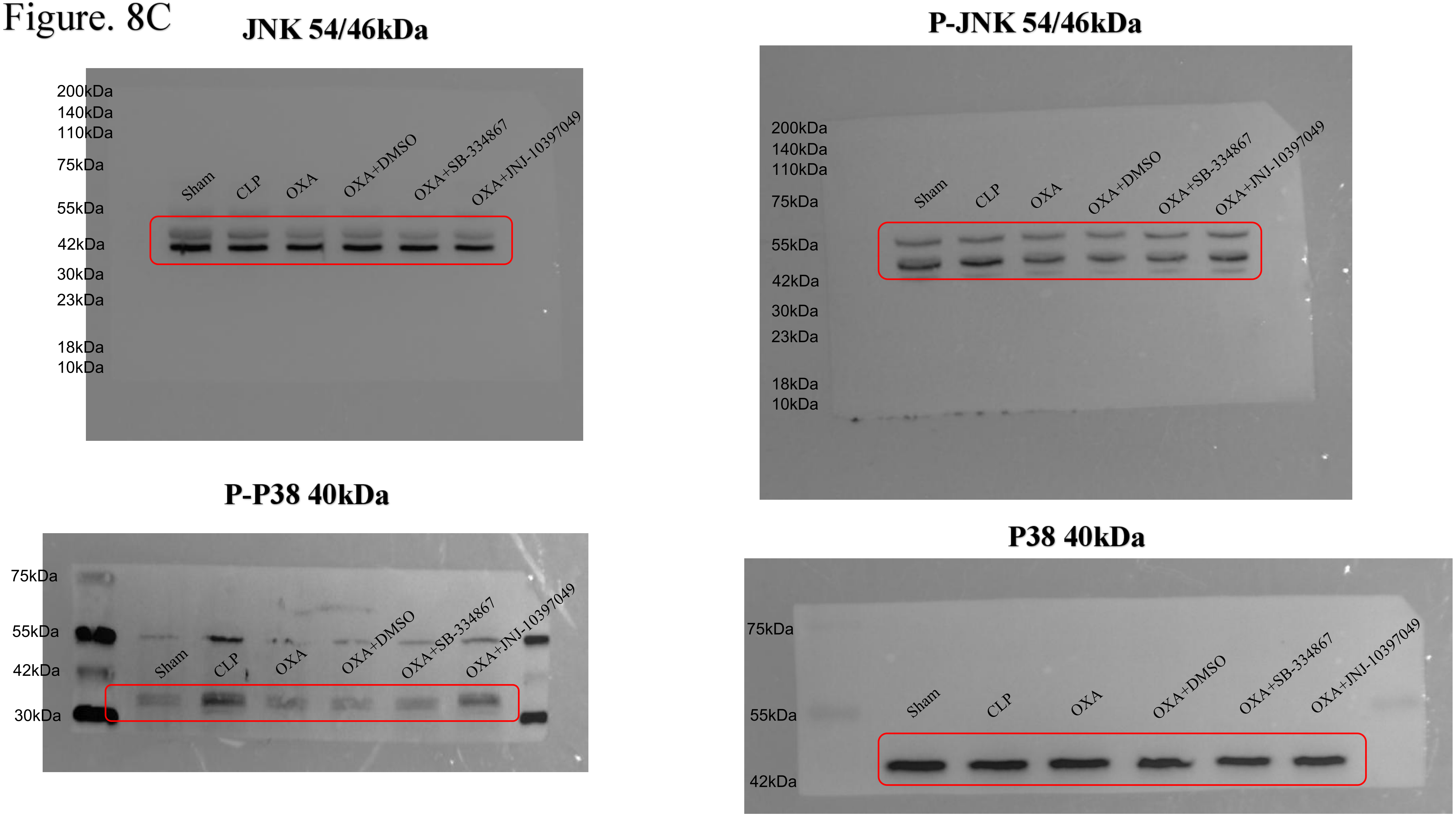
JNK 54/46kDa
200kDa
140kDa
110kDa
75kDa
55kDa
42kDa
30kDa
23kDa
18kDa
10kDa
Sham
CLP
OXA
OXA+DMSO
OXA+SB-334867
OXA+JNJ-10397049
P-JNK 54/46kDa
200kDa
140kDa
110kDa
75kDa
55kDa
42kDa
30kDa
23kDa
18kDa
10kDa
Sham
CLP
OXA
OXA+DMSO
OXA+SB-334867
OXA+JNJ-10397049
P-P38 40kDa
75kDa
55kDa
42kDa
30kDa
Sham
CLP
OXA
OXA+DMSO
OXA+SB-334867
OXA+JNJ-10397049
P38 40kDa
75kDa
55kDa
42kDa
Sham
CLP
OXA
OXA+DMSO
OXA+SB-334867
OXA+JNJ-10397049

Figure. 8C

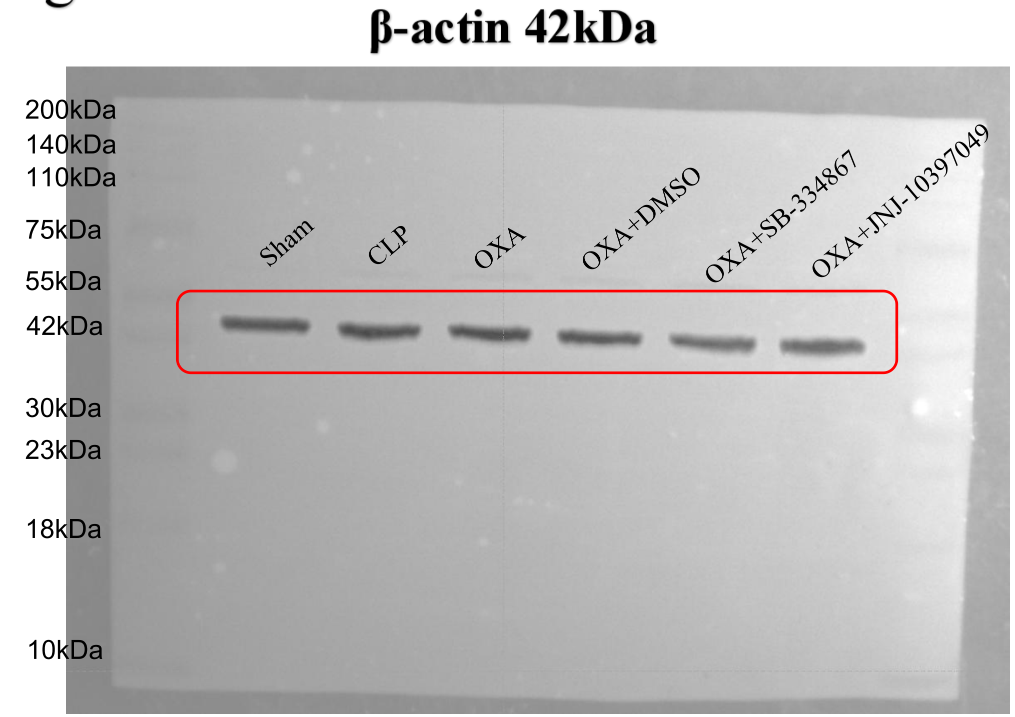
β-actin 42kDa
200kDa
140kDa
110kDa
75kDa
55kDa
42kDa
30kDa
23kDa
18kDa
10kDa
Sham
CLP
OXA
OXA+DMSO
OXA+SB-334867
OXA+JNJ-10397049

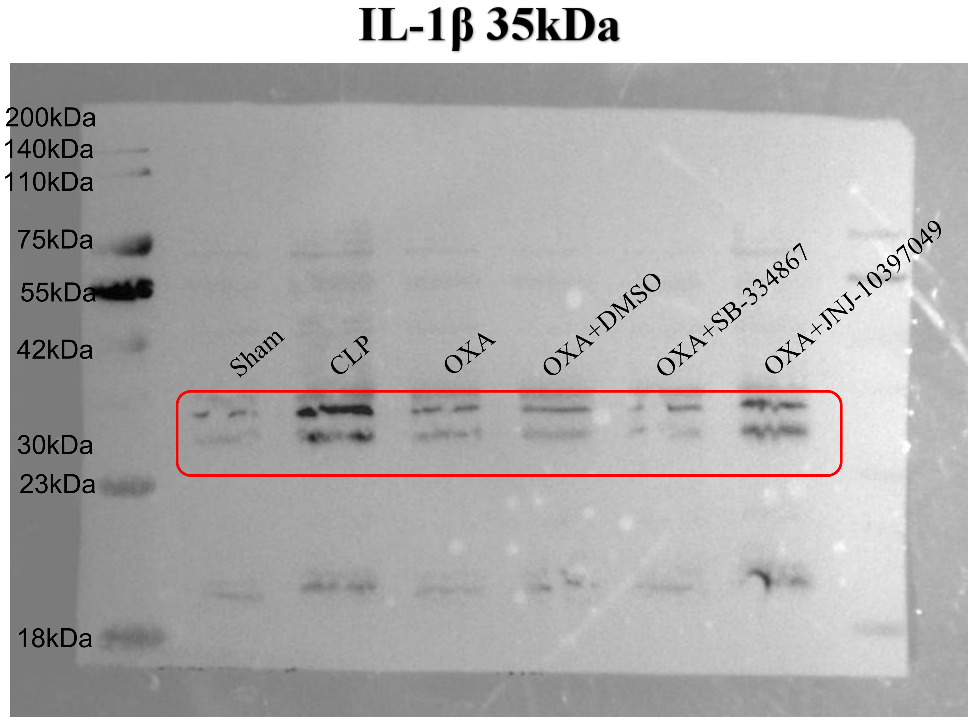
IL-1β 35kDa
200kDa
140kDa
110kDa
75kDa
55kDa
42kDa
30kDa
23kDa
18kDa
Sham
CLP
OXA
OXA+DMSO
OXA+SB-334867
OXA+JNJ-10397049

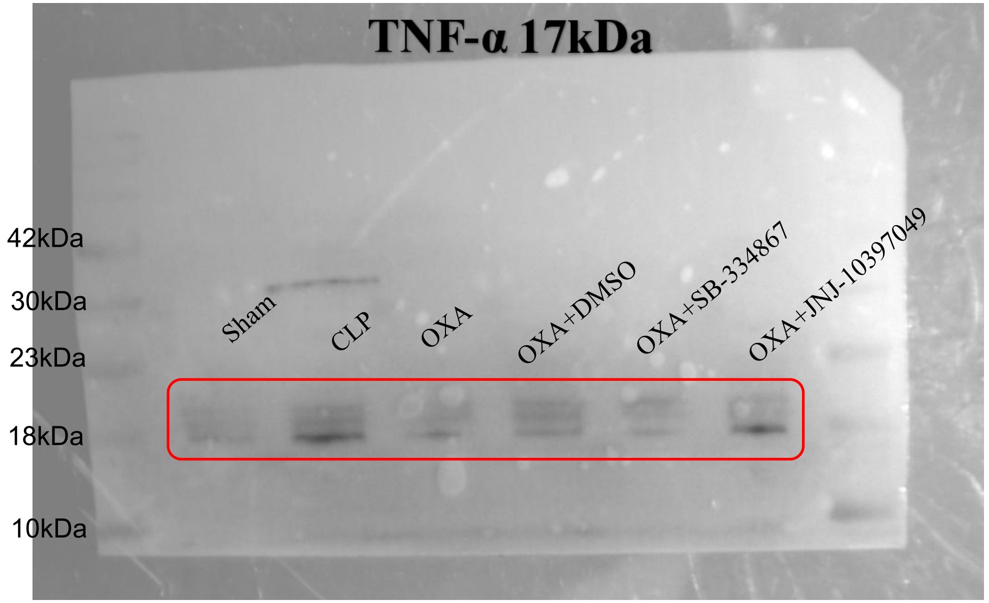
TNF-α 17kDa
42kDa
30kDa
23kDa
18kDa
10kDa
Sham
CLP
OXA
OXA+DMSO
OXA+SB-334867
OXA+JNJ-10397049
