# Supplementary material for: Therapeutic effects of orexin-A in sepsis-associated encephalopathy in mice
Source: J Neuroinflammation. 2024 May 17;21:131. doi: 10.1186/s12974-024-03111-w (PMC11102217; doi:10.1186/s12974-024-03111-w)
Supplement: Supplementary file 2 — Supplementary Material 2 [file 12974_2024_3111_MOESM2_ESM.pdf]

Supplementary files

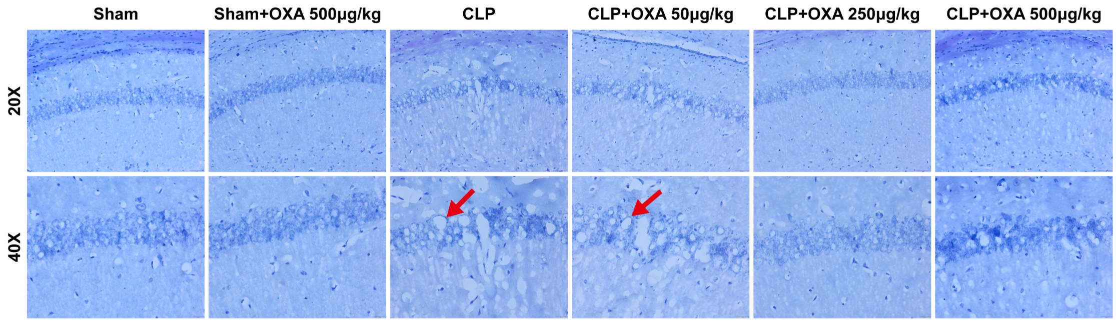

Figure S1. Nissl staining shows the morphology of hippocampal CA1 vertebral neurons. The CLP and OXA low dose group (CLP+OXA 50 μg/kg) showed significant vacuolization and irregular morphology, in which the Nissl body was decreasing or dissolving when observed (red arrow); the medium dose group (CLP+OXA 250 μg/kg) and the high-dose group (CLP+OXA 500 μg/kg) showed recovery of neuronal morphology in the hippocampal CA1 area. n = 3 per group. Data are presented as mean ± SEM. *$p < 0.05$, **$p < 0.01$, ***$p < 0.001$, ****$P < 0.0001$. Scale bar = 50 μm (the upper part of Figure S1.). Scale bar = 20 μm (the lower part of Figure S1).

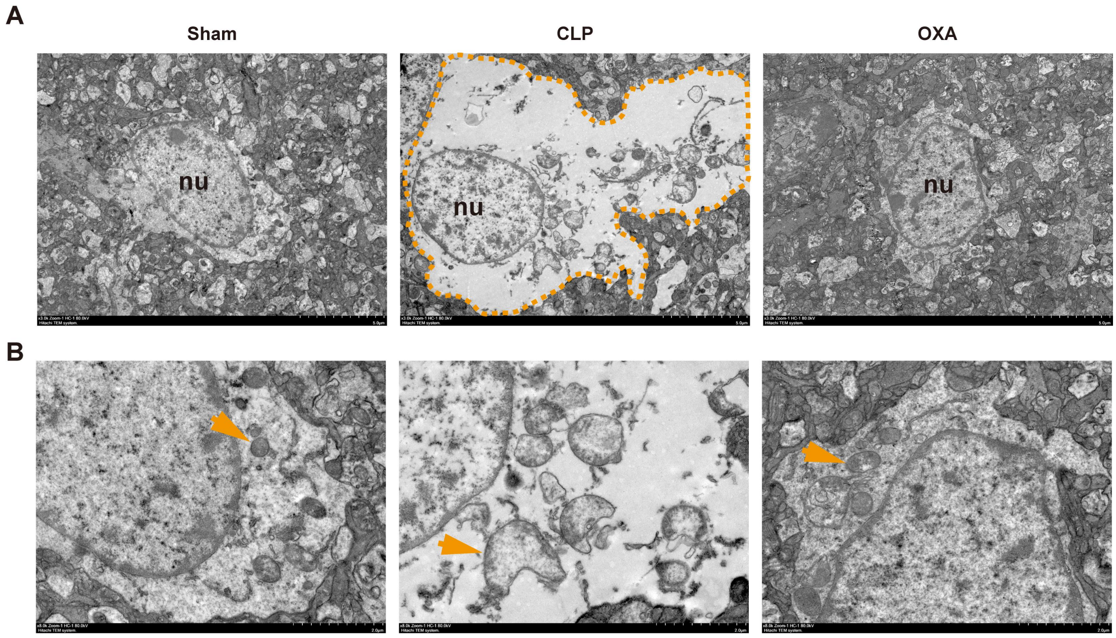

Figure S2. OXA ameliorates ultrastructural damage in astrocytes. **A** Representative images of astrocytes. *n* = 4 per group. Scale bar = 5 μm. nu: nucleus. Yellow dotted area: edematous region of the cytoplasm of astrocytes from the CLP group. **B** is a magnified view of A. Yellow arrows: mitochondria in organelles. Scale bar = 2 μm.
